# Pseudomonadal itaconate degradation gene cluster encodes enzymes for methylsuccinate utilization

**DOI:** 10.1038/s42003-025-08538-2

**Published:** 2025-07-24

**Authors:** Lena Gonner, Eric A. Cassens, Simone König, Ivan A. Berg

**Affiliations:** 1 Institute for Molecular Microbiology and Biotechnology, University of Münster, Münster, Germany; 2 Core Unit Proteomics, Interdisciplinary Center for Clinical Research, Medical Faculty, University of Münster, Münster, Germany

**Keywords:** Bacterial physiology, Bacteriology, Metabolism, Enzyme mechanisms

## Abstract

Branched-chain C_5_-dicarboxylic acids (e.g., citramalate, mesaconate or methylsuccinate) and their CoA-esters are important intermediates in bacterial metabolism, while itaconate is an antimicrobial agent, a potent immunomodulator and a growth substrate for many bacteria. The itaconate degradation pathway consists of three reactions catalyzed by itaconate CoA transferase, itaconyl-CoA hydratase and (*S*)-citramalyl-CoA lyase encoded in a cluster, which in saprophytic bacteria contains two additional genes for a putative acyl-CoA dehydrogenase and a protein of the MmgE/PrpD family. Here, we heterologously produced the corresponding proteins from *Cupriavidus necator* and *Pseudomonas aeruginosa* and showed that they catalyze the (*RS*)-methylsuccinyl-C4-CoA dehydrogenase and an (*S*)-(*R*)-methylsuccinate isomerase reaction, respectively. Together with itaconate CoA transferase, which is highly active with (*R*)-methylsuccinate but has low activity with (*S*)-methylsuccinate, these enzymes allow the utilization of both stereoisomers of methylsuccinate. Our bioinformatic analysis revealed that 1.6% of the sequenced prokaryotes (mainly Betaproteobacteria) possess an identified methylsuccinate isomerase. Analysis of the conserved amino acids of methylsuccinate isomerase and other MmgE/PrpD proteins suggests that they share a common catalytic mechanism via the formation of an enolate intermediate. The presence of specific methylsuccinate utilization genes in the itaconate degradation cluster, which is widespread in saprophytic bacteria, suggests the importance of methylsuccinate in the environment.

## Abstract (web summary)

The identification and characterization of an (*RS*)-methylsuccinyl-C4-CoA dehydrogenase and an (*S*)-(*R*)-methylsuccinate isomerase belonging to the MmgE/PrpD proteins reveals the existence of a widespread methylsuccinate degradation pathway in bacteria.

## Introduction

Branched-chain C_5_-dicarboxylic acids (e.g., citramalate, mesaconate, methylsuccinate, ethylmalonate) and their CoA-esters are important intermediates in bacterial metabolism, including various autotrophic, fermentation, and anaplerotic pathways^1–8^. Furthermore, itaconate (methylene succinate) is a product of the overflow metabolism of soil fungi, an antimicrobial agent, a potent immunomodulator that regulates macrophage inflammation, and a promising platform chemical ranked among the top 12 building block chemicals^9–15^. Itaconate is also a substrate for many bacteria that are able to detoxify it and use it as a carbon source. Being produced by soil and marine microorganisms, as well as in macrophages and monocytes^9–12,16–18^, itaconate can be degraded by many soil bacteria (e.g., *Paraburkholderia xenovorans*, *Cupriavidus necator*)^19–21^, pathogens (e.g., *Yersinia pestis*, *Salmonella* *Typhimurium*, *Pseudomonas aeruginosa*)^20^, and fungi^22^. The corresponding pathway consists of itaconate activation to itaconyl-CoA, its hydration and isomerization to (*S*)-citramalyl-CoA, and cleavage into acetyl-CoA and pyruvate^19,20^.

**Fig. 1:**
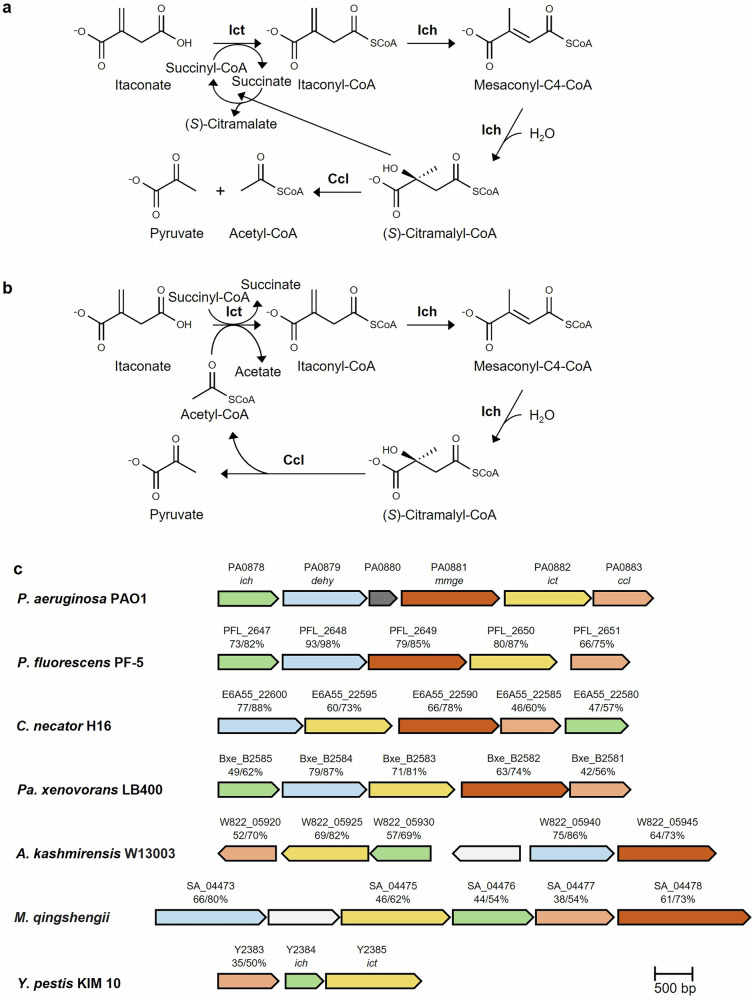
Itaconate degradation pathway. The variants of the pathway functioning in *P. aeruginosa* (**a**), *Y. pestis* (**b**), and the corresponding itaconate degradation cluster found in different pseudomonads (**c**), are shown. The identity/similarity of the genes to the corresponding *P. aeruginosa* PAO1 genes is given in %, the genes are colored according to their homology. *ich* itaconyl-CoA hydratase, *mcd* putative acyl-CoA dehydrogenase, *mmgE* MmgE/PrpD family protein, *ict* itaconate CoA transferase, *ccl* (*S*)-citramalyl-CoA lyase. The *P. aeruginosa* itaconate degradation cluster also encodes a gene annotated as a ring-cleaving dioxygenase (PA0880, shown in dark grey), which has been proposed to function as a 3-hydroxyparaconate lactonase^23^. The full locus tag of *Mesorhizobium qingshengii* (SAMN02927914_) was shortened (SA_). *A. kashmirensis, Advenella kashmirensis*.

The itaconate degradation pathway exists in two convergently evolved variants, which use nonhomologous or only distantly homologous enzymes, with CoA transferases having different substrate spectra^20^. In *Y. pestis*, the enzyme was highly specific for itaconate, but could use acetyl-CoA as a CoA donor in addition to succinyl-CoA (Fig. 1b). As a consequence, acetyl-CoA (the product of itaconate degradation) served as the CoA donor for itaconate activation, making itaconate degradation independent of the initial concentration of succinyl-CoA (Fig. 1b). In contrast, itaconate CoA transferase from *P. aeruginosa* was succinyl-CoA-dependent and could not use acetyl-CoA as a CoA-donor. However, this enzyme was able to activate methylsuccinate and (*S*)-citramalate and catalyzed CoA transfer from (*S*)-citramalyl-CoA to itaconate. Therefore, together with itaconyl-CoA hydratase, this enzyme catalyzed the rapid conversion of itaconate to nontoxic citramalate, which was independent of the initial concentration of succinyl-CoA^20^ (Fig. 1a).

Besides genes involved in itaconate degradation, the *P. aeruginosa* gene cluster contained genes encoding a glyoxalase family protein, a putative acyl-CoA dehydrogenase and an MmgE/PrpD family protein (Fig. 1c). These two proteins are normally present in the itaconate degradation operon of many nonpathogenic soil pseudomonads like *Pa. xenovorans* or *C. necator* (Fig. 1c). We hypothesized that these genes might be responsible for the channeling of citramalate, mesaconate, and methylsuccinate in the pathway^20^. Specifically, we proposed that the putative acyl-CoA dehydrogenase might catalyze the methylsuccinyl-CoA dehydrogenase reaction, whereas the MmgE/PrpD family protein might be responsible for the conversion of mesaconate to (*S*)-citramalate. The latter hypothesis was soon rejected, as we were able to show that class I fumarases catalyze the formation of (*S*)-citramalate from mesaconate^21^. More recently, de Witt et al.^23^ studied the function of the enzymes of the *P. aeruginosa* cluster by heterologously transferring it to *P. putida*, which does not naturally possess this cluster, and analyzing its growth phenotype. The results of their study were consistent with the putative acyl-CoA dehydrogenase functioning as a methylsuccinyl-CoA dehydrogenase, while the (*S*)-(*R*)-methylsuccinate isomerase activity of the MmgE/PrpD family protein was proposed^23^. In addition, their analysis suggested that the activity of itaconate CoA transferase might be limited to (*R*)-methylsuccinate, explaining the requirement of the (*S*)-(*R*)-methylsuccinate isomerase for (*S*)-methylsuccinate degradation.

In this work, we experimentally investigated the functions of the itaconate degradation cluster and found that it is responsible for the degradation of methylsuccinate as well, with the MmgE/PrpD family protein being indeed (*S*)-(*R*)-methylsuccinate isomerase and acyl-CoA dehydrogenase catalyzing the conversion of both (*R*)- and (*S*)-methylsuccinyl-C4-CoA to mesaconyl-C4-CoA. The presence of two enzymes specific for the degradation of methylsuccinate in the itaconate degradation cluster, which is widespread in soil bacteria, suggests that methylsuccinate may be an important and abundant metabolite in soils.

## Results

### Genome analysis of *C. necator*

The degradation of itaconate requires three enzymes: itaconate CoA transferase, which catalyzes its activation to itaconyl-CoA; itaconyl-CoA hydratase, which converts it to (*S*)-citramalyl-CoA; and (*S*)-citramalyl-CoA lyase, which yields acetyl-CoA and pyruvate. However, itaconate can also be activated by succinyl-CoA synthetase and the itaconate CoA transferase gene is absent from the itaconate degradation cluster in some bacteria^20,21,23,24^. Therefore, only itaconyl-CoA hydratase and (*S*)-citramalyl-CoA lyase can be considered as specific enzymes for the pathway.

The *C. necator* genome contains two homologs of itaconyl-CoA hydratase and (*S*)-citramalyl-CoA lyase genes. In one case, they are co-localized in the genome as part of the cluster homologous to the itaconate degradation cluster of *P. aeruginosa* and *Pa. xenovorans* (E6A55_22580-22600; Fig. 1c). In contrast, the second itaconyl-CoA hydratase homolog was co-localized with a CoA transferase gene and an adenylosuccinate lyase family protein, whereas no citramalyl-CoA lyase homolog was found in its vicinity, suggesting its function in a different pathway (Supplementary Fig. 1). Similarly, the gene encoding the second (*S*)-citramalyl-CoA lyase homolog was co-localized with a CoA transferase gene, but not with an itaconyl-CoA hydratase homolog, suggesting that it is not involved in itaconate assimilation. Therefore, the genomic analysis provides strong support of the identification of E6A55_22580-22600 as the itaconate degradation cluster in *C. necator*.

The *C. necator* genome does not possess a homologue for the branched short-chain dicarboxylate transporter identified in *P. aeruginosa*^23^. While a gene annotated as a tripartite tricarboxylate transporter substrate binding protein (E6A55_22575) is encoded in close proximity to the *C. necator* itaconate degradation cluster, its function is unknown.

### Growth of *C. necator* on itaconate, (*S*)- and (*R*)-methylsuccinate

The presence of the itaconate degradation gene cluster in the *C. necator* genome (Fig. 1c) suggests that this bacterium may be able to utilize itaconate and related compounds. Indeed, *C. necator* grew on itaconate as the sole carbon and energy source, although growth was slower and the final OD was lower than on succinate (Supplementary Fig. 2). *C. necator* also grew on (*S*)- and (*R*)-methylsuccinate, with growth on the (*R*)-stereoisomer of methylsuccinate being slightly better than on the (*S*)-stereoisomer (Supplementary Fig. 2).

### Enzyme activities in *C. necator*

**Fig. 2:**
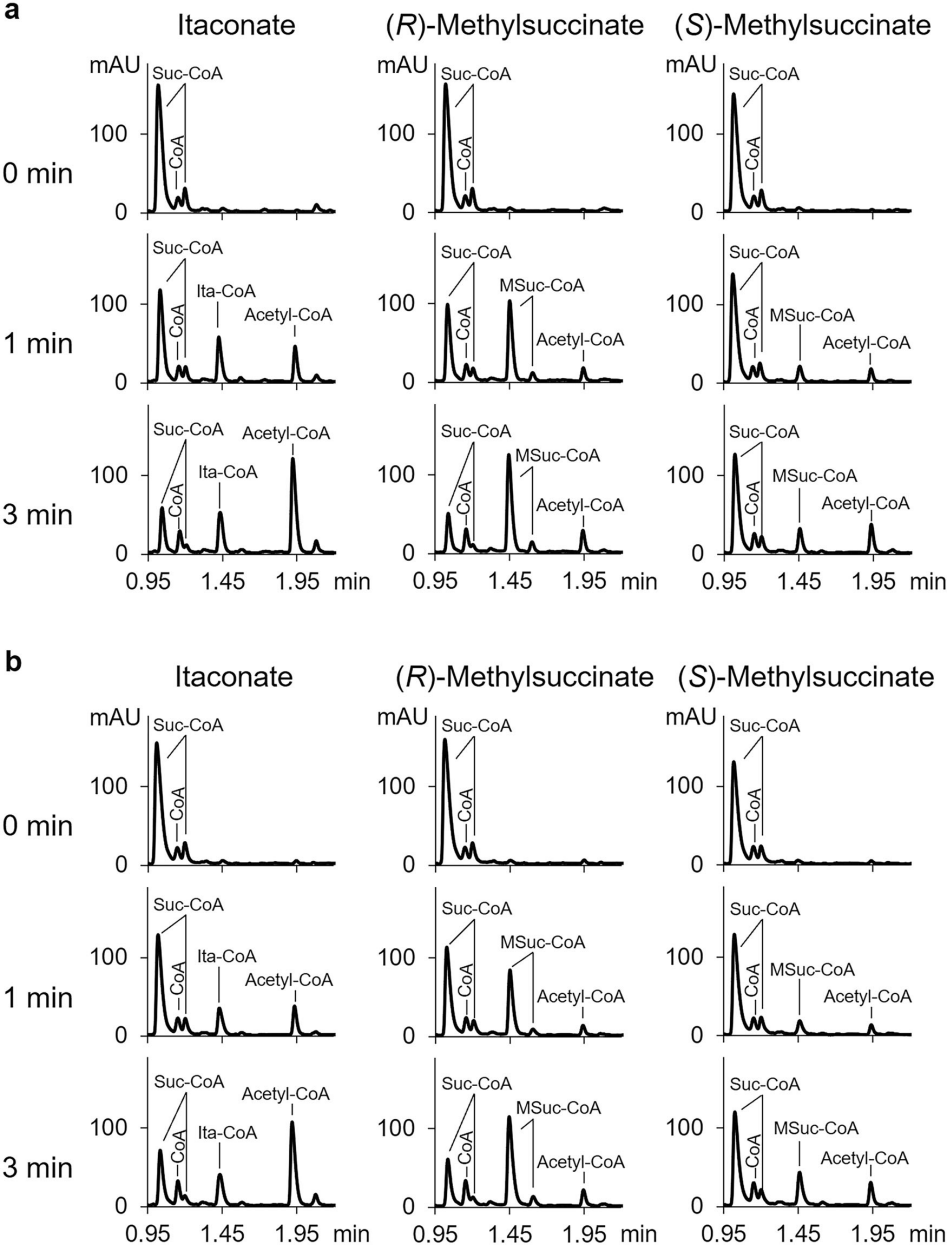
Conversion of itaconate, (*S*)-methylsuccinate and (*R*)-methylsuccinate by cell extracts of *C. necator*. Conversions for cell extracts of **a** itaconate- and **b** (*RS*)-methylsuccinate-grown *C. necator* are shown. The experiment was started by the addition of succinyl-CoA, and the samples after 0, 1, and 3 min of incubation were analysed by reversed phase C18 UHPLC to follow CoA-esters at 260 nm. Note that in a separation method we use for CoA-esters, they form double peaks, where the second, smaller peak is 0.15 min away from the first peak. The protein concentrations for the experiments with extracts of itaconate- and methylsuccinate-grown cells were 1.5 mg protein ml^−1^, respectively. The ordinate axis shows absorption at 260 nm (arbitrary units).

Cell extracts of itaconate- and (*RS*)-methylsuccinate-grown *C. necator* were able to convert itaconate into acetyl-CoA and pyruvate in the presence of succinyl-CoA with similar activities (Fig. 2, Table 1). Itaconyl-CoA was detected as an intermediate in this conversion (Fig. 2). Extracts of itaconate- and (*RS*)-methylsuccinate-grown cells converted both stereoisomers of methylsuccinate to acetyl-CoA with methylsuccinyl-CoA as an intermediate (Fig. 2 and Table 1), whereas the activity of these conversions in succinate-grown cell was lower (Table 1).

**Table 1:** Activities of itaconate and methylsuccinate conversions in the presence of succinyl-CoA in extracts of *C. necator* cells grown on different substrates (in µmol min^−1^ mg^−1^ protein)

Reaction/reaction substrates	Growth substrate		
	Succinate	Itaconate	Methylsuccinate
Itaconate CoA transferase	0.12 ± 0.05 (*n* = 4)	0.27 ± 0.07 (*n* = 9)	0.30 ± 0.09 (*n* = 8)
Itaconate conversion to acetyl-CoA	0.01 ± 0.005 (*n* = 4)	0.11 ± 0.03 (*n* = 9)	0.17 ± 0.06 (*n* = 8)
(*S*)-Methylsuccinate CoA transferase	0.03 ± 0.004 (*n* = 3)	0.11 ± 0.04 (*n* = 7)	0.13 ± 0.07 (*n* = 9)
(*S*)-Methylsuccinate conversion to acetyl-CoA	0.01 ± 0.001 (*n* = 3)	0.03 ± 0.01 (*n* = 6)	0.04 ± 0.01 (*n* = 9)
(*R*)-Methylsuccinate CoA transferase	0.08 ± 0.04 (*n* = 4)	0.31 ± 0.06 (*n* = 8)	0.30 ± 0.06 (*n* = 8)
(*R*)-Methylsuccinate conversion to acetyl-CoA	0.02 ± 0.01 (*n* = 3)	0.03 ± 0.01 (*n* = 7)	0.035 ± 0.003 (*n* = 8)

For the calculation of CoA transferase activities, the amounts of acetyl-CoA and the corresponding intermediates were summed up. Data are mean ± s.d. and the number of replicates (*n*) is shown. The concentrations of itaconate, (*S*)- and (*R*)-methylsuccinate were 5 mM, the concentration of succinyl-CoA was 1 mM.

### Proteomic analysis

To further study the degradation of itaconate and methylsuccinate in *C. necator*, we performed a proteomic analysis comparing the proteome of cells grown on itaconate and (*RS*)-methylsuccinate with that of cells grown on succinate (Supplementary Table 1 and Supplementary Data 1, 2). All proteins encoded in the itaconate degradation cluster were detected in cell extracts and were up-regulated during growth on itaconate and methylsuccinate. Although the up-regulation for some of the proteins was low (1.2–2.3 fold), the dehydrogenase from the cluster was strongly up-regulated (83 fold in itaconate-grown cells). Therefore, it was sufficient to explain the differences in the activities of the conversions of itaconate and methylsuccinate to acetyl-CoA in *C. necator* cell extracts (Table 1 and Supplementary Table 1), considering that this process consists of three (itaconate metabolism) or four (methylsuccinate) reactions. However, the proteomic detection of itaconate CoA transferase was not reliable, as only one unique peptide was identified for this protein, probably due to the presence of several CoA transferase homologs in the *C. necator* genome. Interestingly, the second itaconyl-CoA hydratase and (*S*)-citramalyl-CoA lyase homologs were detected with low spectral intensity, indicating low abundance (Supplementary Data 1 and 2), confirming their potential involvement in other metabolic processes.

### Characterization of itaconate CoA transferase

The degradation of itaconate and methylsuccinate starts with their activation to the corresponding CoA-esters (Fig. 2). Therefore, we first characterized the *C. necator* itaconate CoA transferase homologue (E6A55_22595, Q0K3E5), which shares 60% identity/73% similarity with the *P. aeruginosa* itaconate CoA transferase PA0882. We cloned and heterologously expressed the corresponding gene in *Escherichia coli* using the pET16b vector, which introduces an N-terminal His-tag to the protein. The purified CoA transferase was biochemically characterized (Table 2). The catalytic properties of the *C. necator* protein were similar to those of PA0882^20^. The protein could only use succinyl-CoA for itaconate activation and was active with (*S*)- but not with (*R*)-citramalate. Although the protein could activate both (*R*)- and (*S*)-methylsuccinate, the catalytic efficiency with the (*S*)-stereoisomer was very low (65 vs 0.7 s^−1^ mM^−1^, respectively), suggesting that only its activity with (*R*)-methylsuccinate is of physiological relevance. As the activity with different stereoisomers of methylsuccinate was not tested for the *P. aeruginosa* enzyme, we also expressed it heterologously and tested it with (*S*)- and (*R*)-methylsuccinate. Like the *C. necator* CoA transferase, the *P. aeruginosa* enzyme was highly active with (*R*)-, but not with (*S*)-methylsuccinate, identifying (*R*)-methylsuccinate as its physiological substrate (Table 2). Interestingly, the catalytic efficiency of itaconate CoA transferases from both organisms was higher for (*R*)-methylsuccinate than for itaconate, highlighting the importance of the degradation of this substrate for both organisms, especially for soil *C. necator*, where the catalytic efficiencies differed sevenfold between these two substrates (Table 2).

**Table 2:** Catalytic properties of heterologously produced itaconate CoA transferases from *C. necator* (E6A55_22600) and *P. aeruginosa* (PA0882)

Substrate	*V*_max_ (µmol min^−1^ mg^−1^ protein)	*K*_M_ (mM)	*k*_cat_/*K*_M_ (s^−1^ mM^−1^)
*C. necator* itaconate CoA transferase			
Itaconate	53.2 ± 10.1	4.0 ± 1.7	9.5
Mesaconate	<0.1	NA	NA
Acetate	<0.1	NA	NA
Butyrate	<0.1	NA	NA
Propionate	<0.1	NA	NA
(*S*)-Methylsuccinate	35.6 ± 1.4	35.6 ± 3.2	0.7
(*R*)-Methylsuccinate	82.0 ± 3.4	0.9 ± 0.1	65.2
(*S*)-Citramalate	10.2 ± 0.6	15.6 ± 2.3	0.5
(*R*)-Citramalate	<1	NA	NA
Glutarate	3.2 ± 0.3	37.4 ± 8.9	0.06
(*S*)-Malate	1.1 ± 0.1	42.8 ± 17.2	0.02
(*R*)-Malate	<0.1	NA	NA
Succinyl-CoA	116.3 ± 14.1	0.76 ± 0.2	109.4
Acetyl-CoA	<0.1	NA	NA
Propionyl-CoA	<0.1	NA	NA
Butyryl-CoA	<0.1	NA	NA
*P. aeruginosa* itaconate CoA transferase			
Itaconate	73.0 ± 6.4	0.8 ± 0.3	66.3
(*S*)-Methylsuccinate	54.3 ± 8.6	17.8 ± 8.0	2.2
(*R*)-Methylsuccinate	69.8 ± 5.7	0.6 ± 0.2	88.0

Values shown are the mean values ± s.d. of results from three independent measurements. Measurements were performed with 1 mM succinyl-CoA (for *V*_max_/*K*_M_ determination for carboxylic acids) or with 10 mM itaconate (for *V*_max_/*K*_M_ value determination for CoA-esters).

*NA* not applicable.

### Identification of the MmgE/PrpD family protein as (*S*)-(*R*)-methylsuccinate isomerase

**Fig. 3:**
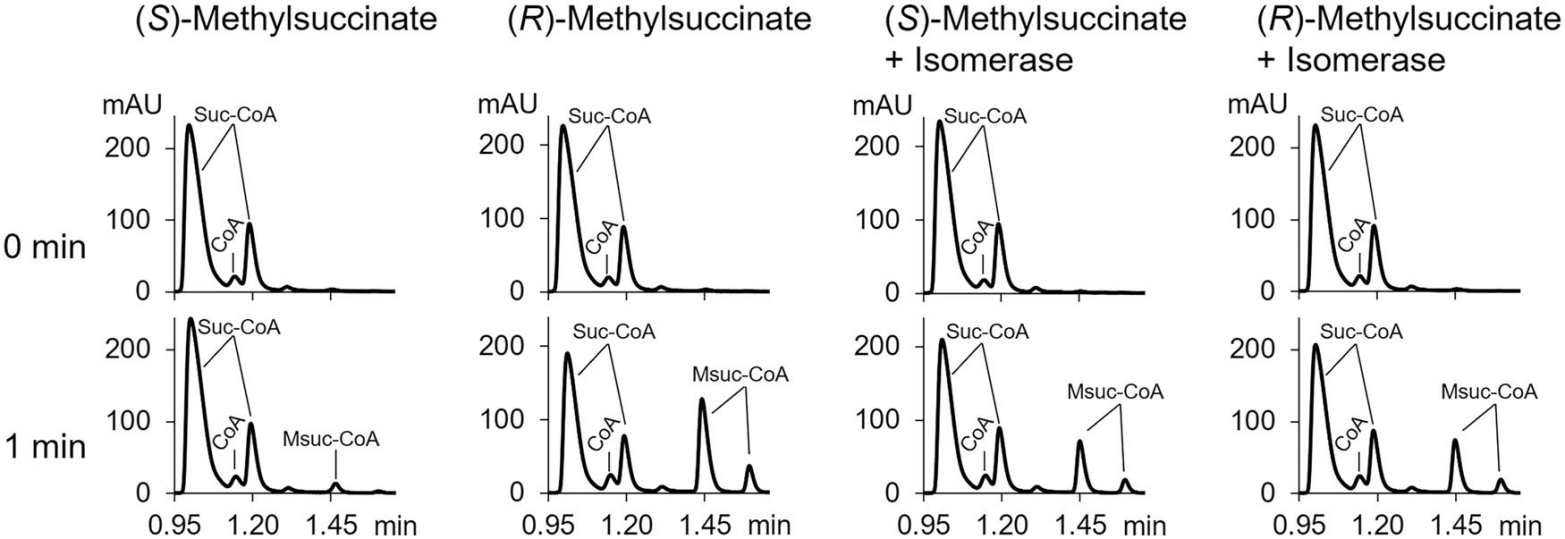
Identification of the *C. necator* MmgE/PrpD family protein as (*S*)-(*R*)-methylsuccinate isomerase. (*S*)-Methylsuccinate and (*R*)-methylsuccinate were converted to the corresponding CoA-ester in the presence of succinyl-CoA and the *C. necator* itaconate CoA transferase (0.22 mg protein ml^−1^) specific for (*R*)-methylsuccinate. The samples incubated for 1 min in the presence of MmgE/PrpD protein (right side, 0.7 mg protein ml^−1^) and in the absence of MmgE/PrpD protein (left side) are shown. The samples were analyzed by reversed phase C18 UHPLC to follow CoA-esters at 260 nm. Note that in a separation method we use for CoA-esters, they form double peaks, where the second, smaller peak is 0.15 min away from the first peak.

Although the purified itaconate CoA transferases preferentially used the (*R*)-stereoisomer of methylsuccinate, the difference in growth on the (*R*)- and (*S*)-stereoisomers and in their conversion to acetyl-CoA in cell extract assays was much smaller (Fig. 2, Table 1 and Supplementary Fig. 2). This suggests the existence of an isomerase that interconverts these two substrates, which had been proposed to be the MmgE/PrpD family protein (E6A55_22590/Q0K3E6 in *C. necator* and PA0881 in *P. aeruginosa*)^23^. We synthesized these two genes, cloned them into the pET16b vector (Supplementary Table 2), expressed them heterologously in *E. coli* and characterized the purified enzymes. To measure enzyme activity, we took advantage of the fact that itaconate CoA transferase is preferentially active with (*R*)-methylsuccinate, and followed the difference in methylsuccinyl-CoA formation after incubation of (*S*)-methylsuccinate with CoA transferase in the presence and in the absence of the MmgE/PrpD family protein (Fig. 3). Indeed, both MmgE/PrpD proteins catalyzed the isomerization of (*S*)-methylsuccinate to (*R*)-methylsuccinate. The specific activities of the isomerases from these two organisms were similar: 72.3 ± 9.8 µmol min^−1^ mg^−1^ protein for the *C. necator* enzyme and 78.7 ± 27.4 µmol min^−1^ mg^−1^ protein for the *P. aeruginosa* isomerase. In addition, we tested the isomerase activity of the *Pa. xenovorans* MmgE/PrpD family protein Bxe_B2582 that we cloned previously^21^: the specific activity of this isomerase was 53.2 ± 19.0 µmol min^−1^ mg^−1^ protein. To check whether the isomerase is also active with methylsuccinyl-CoA, we used the same approach with itaconate CoA transferase but measured the formation of succinyl-CoA from (*S*)-methylsuccinyl-CoA and succinate with and without the *C. necator* isomerase. Since the addition of isomerase had no effect on succinyl-CoA formation, we concluded that the enzyme is only active with carboxylic acids and not with their CoA-esters.

To summarize, we demonstrated that the MmgE/PrpD family protein from the itaconate degradation cluster is indeed an (*S*)-(*R*)-methylsuccinate isomerase. This protein showed no (*S*)-(*R*)-citramalate isomerase activity (detection limit: 0.01 µmol min^−^^1^ mg^−^^1^ protein), and no activity could be detected with itaconate, citraconate, mesaconate, *cis*-aconitate, *trans*-aconitate (detection limit: 0.005 µmol min^−^^1^ mg^−^^1^), or (*R*)-malate (detection limit: 0.003 µmol min^−^^1^ mg^−^^1^). We conclude that the isomerase is specific to methylsuccinate.

### Identification of acyl-CoA dehydrogenase as (*RS*)-methylsuccinyl-CoA dehydrogenase

The next enzyme required for methylsuccinate degradation is a dehydrogenase that converts methylsuccinyl-CoA to mesaconyl-C4-CoA, which was hypothesized to be the acyl-CoA dehydrogenase from the itaconate degradation cluster^20,23^. We cloned the corresponding gene from *C. necator* (E6A55_22600/Q0K3E4) into the pET23b vector, heterologously expressed it and characterized the product. We also synthesized the *P. aeruginosa* dehydrogenase gene PA0879, cloned it in the pET23a vector, expressed it in *E. coli*, purified the protein, and used it for biochemical analysis. The proteins were active with both stereoisomers of methylsuccinyl-CoA with similar catalytic efficiencies, showing that the enzyme is not stereoselective (Table 3). The activity was measured with the artificial electron acceptor phenazine methosulfate (PMS), whereas no activity was detected with NAD(P), ferricenium and cytochrome *c* (detection limit: 0.025 µmol min^−^^1^ mg^−^^1^). The dehydrogenase had been annotated as a flavin enzyme, and purification of the dehydrogenase from *C. necator* and *P. aeruginosa* in an active form was only possible when FAD was added to the purification buffer, indicating that FAD is only loosely bound to the protein.

**Table 3:** Catalytic properties of heterologously produced methylsuccinyl-CoA dehydrogenase from *C. necator* (E6A55_22600) and *P. aeruginosa* (PAO879)

Enzyme/parameter	(*S*)-Methylsuccinyl-CoA	(*R*)-Methylsuccinyl-CoA
*C. necator* methylsuccinyl-C4-CoA dehydrogenase		
*V*_max_, µmol min^−1^ mg^−1^ protein	0.69 ± 0.08	0.72 ± 0.05
* K*_M_, mM	0.42 ± 0.14	0.36 ± 0.08
* k*_cat_/*K*_M_, s^−1^ mM^−1^	1.2	1.5
*P. aeruginosa* methylsuccinyl-C4-CoA dehydrogenase		
*V*_max_, µmol min^−1^ mg^−1^ protein	2.80 ± 0.36	3.38 ± 0.36
* K*_M_, mM	0.47 ± 0.13	0.59 ± 0.14
* k*_cat_/*K*_M_, s^−1^ mM^−1^	4.3	4.1

The values shown are mean values ± s.d. of results from three independent measurement.

**Fig. 4:**
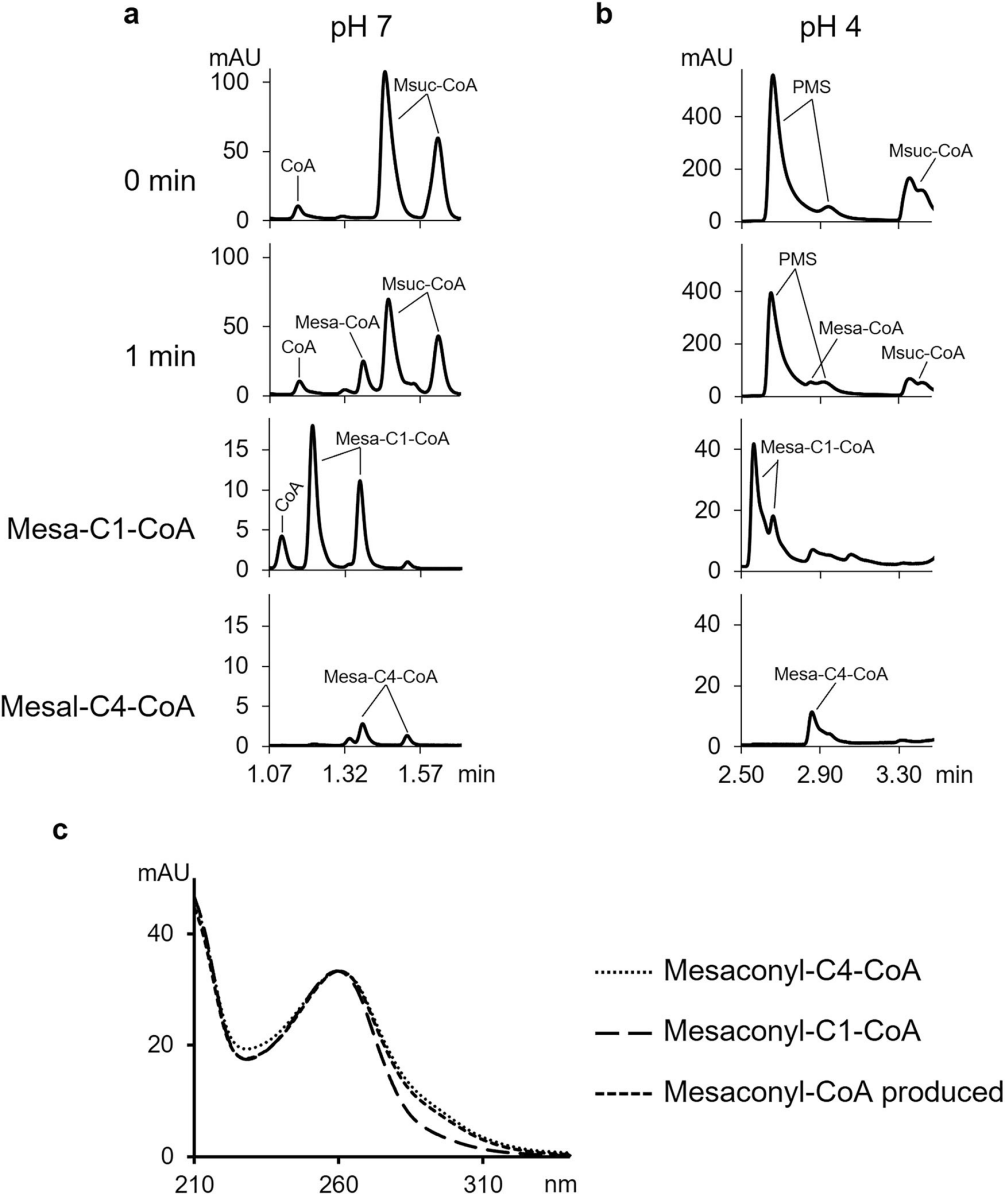
Identification of mesaconyl-C4-CoA as the product of the methylsuccinyl-CoA dehydrogenase reaction catalyzed by the *C. necator* enzyme. A dehydrogenase assay was performed with (*RS*)-methylsuccinyl-CoA, and the product of the reaction (mesaconyl-CoA, Mesa-CoA) was compared with the standards mesaconyl-C1-CoA and mesaconyl-C4-CoA. The samples were analysed by reversed phase C18 UHPLC to follow CoA-esters at 260 nm. Note that in a separation method we use for CoA-esters, they form double peaks, where the second, smaller peak is 0.15 min away from the first peak. As the retention time of CoA-esters of dicarboxylic acids is strongly dependent on the pH of the UHPLC buffer, the buffers at pH 7 (**a**) and pH 4 (**b**) were used. The spectra of the produced mesaconyl-CoA, mesaconyl-C1-CoA and mesaconyl-C4-CoA are shown in (**c**). The concentration of dehydrogenase in the reaction mixture was 1.0 mg protein ml^−1^.

To further characterize the enzyme, we tested whether methylsuccinyl-C1-CoA or methylsuccinyl-C4-CoA was the substrate of the dehydrogenase. To do this, we studied which of the isomers of mesaconyl-CoA (C1- or C4-CoA-ester) is produced from the methylsuccinyl-CoA synthesized from (*RS*)-methylsuccinate by a mixed anhydride method^25^, which produces the mixture of C1- and C4-CoA-esters^3^. This identification is possible because mesaconyl-C1-CoA and mesaconyl-C4-CoA have different retention times in HPLC and slightly different UV spectra between 270 and 320 nm (Fig. 4; in ref. ^3^). We analysed the reaction products in ultra-high performance liquid chromatography (UHPLC) using two different HPLC buffers, at pH 7 and at pH 4, as the retention times of CoA-esters of dicarboxylic acids are significantly different depending on whether the free carboxyl group of a CoA-ester is protonated or not. Our results showed that the retention times and spectrum of mesaconyl-CoA produced in the methylsuccinyl-CoA dehydrogenase reaction were identical to the retention times and spectrum of mesaconyl-C4-CoA (Fig. 4). Therefore, we conclude that the acyl-CoA dehydrogenase from the itaconate degradation cluster is (*RS*)-methylsuccinyl-C4-CoA dehydrogenase.

When the identified methylsuccinyl-CoA dehydrogenase was incubated with (*R*)-methylsuccinyl-CoA produced enzymatically using *C. necator* itaconate CoA transferase, over 90% of the methylsuccinyl-CoA was converted to mesaconyl-C4-CoA, indicating that the itaconate CoA transferase produces only methylsuccinyl-C4-CoA. Obviously, this specificity is advantageous for the cell, since methylsuccinyl-C1-CoA would be a dead-end product for *C. necator*.

### Distribution of the (*S*)-(*R*)-methylsuccinate isomerase in microbial genomes

**Fig. 5:**
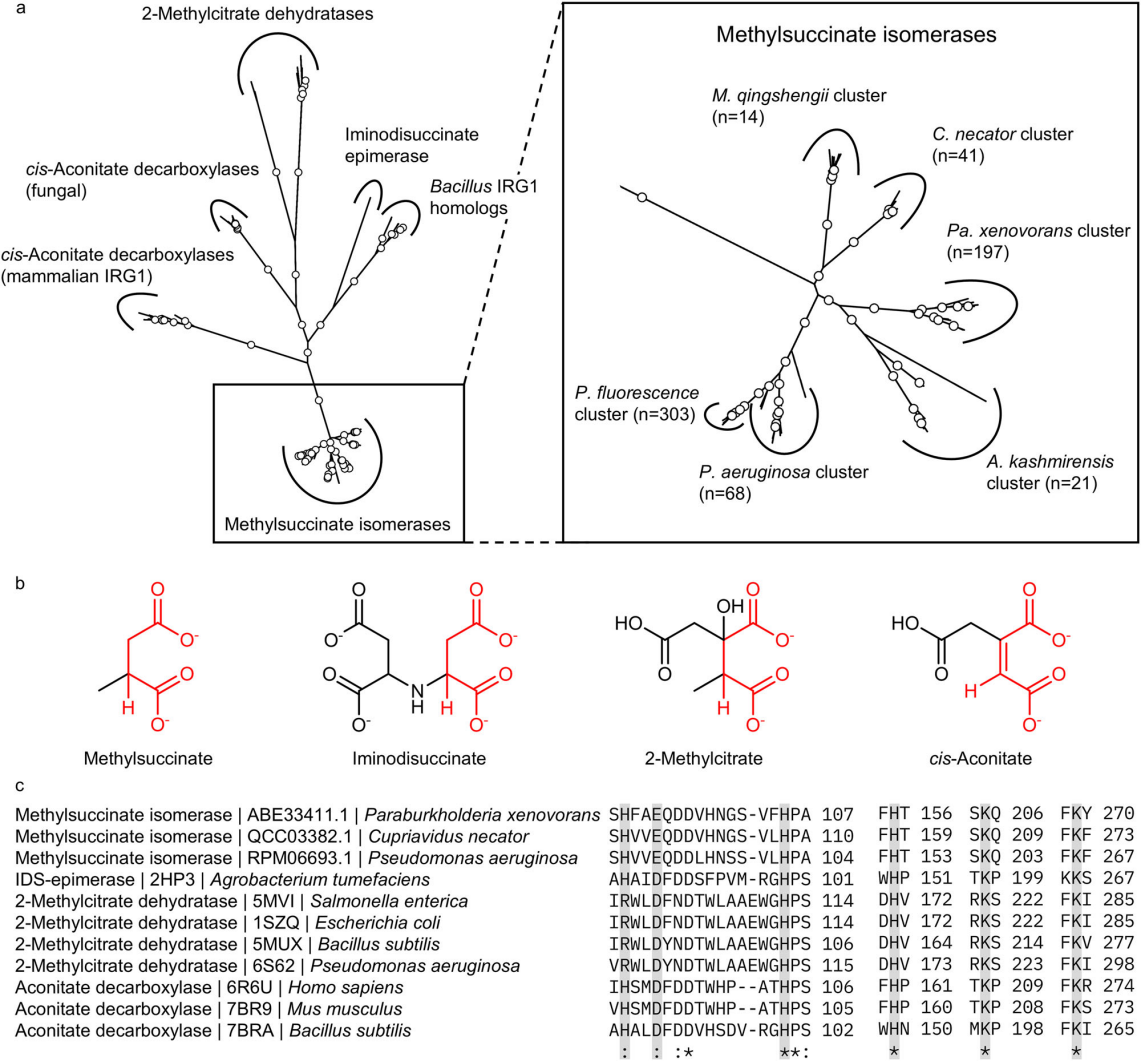
MmgE/PrpD family proteins. Phylogeny (**a**), substrates (**b**) and conserved active site residues (**c**) of the MmgE/PrpD family proteins. Sequences were aligned using MUSCLE and the phylogenetic tree was constructed using the maximum likelihood method. Clades with bootstrap values >0.7 are indicated with white dots. Where possible, ten representative sequences were used for each known family member (Supplementary Table 5). For iminodisuccinate epimerase, only one sequence could be identified using the BlastP search. The *Bacillus subtilis* IRG1 homolog, previously described as a *cis*-aconitate decarboxylase with no evidence of enzymatic activity^27^, had only a low degree of similarity to both fungal and mammalian *cis*-aconitate decarboxylases and most likely catalyzes a different, unknown reaction. Ten representatives were selected for each differently organized gene cluster containing all genes encoding enzymes for the conversion of methylsuccinate to acetyl-CoA and pyruvate, and the total number of gene clusters identified in GTDB is shown as *n*. The substrates of all known MmgE/PrpD family members (**b**) share a four-carbon dicarboxylate substructure highlighted in red. The amino acids that coordinate this shared substructure (highlighted by grey bars) in *cis*-aconitate decarboxylase^40^, IDS epimerase^37^, and methylsuccinate isomerase are conserved in all members of this protein family. For simplicity, only MmgE/PrpD family members with known crystal structures and three methylsuccinate isomerases are shown in alignment (**c**).

To study the distribution and abundance of the identified methylsuccinate isomerase, a hidden Markov model (HMM) of the protein was constructed. We used this model to search against a dereplicated form of the Genome Taxonomy Database (Release 220 GTDB) containing 113,104 prokaryotic genomes^26^. This search identified 50,344 significant hits (*E*-value < 1e-05) in 21,713 unique organisms, showing that 19.2% of the unique species in the GTDB have at least one MmgE/PrpD homolog. The identified homologs included not only methylsuccinate isomerases, but also other known members of the MmgE/PrpD protein family, but mostly uncharacterized proteins (Fig. 5a). Interestingly, the *Bacillus subtilis* MmgE/PrpD homolog, described as an IRG1 protein^27^, did not fall into the clusters containing *cis*-aconitate decarboxylases (neither fungal nor mammalian IRG1) and probably has a different, as yet undetermined function.

Based on the scores obtained from the HMM search and the analysis of the genomic region flanking the gene encoding the target protein, we found that the genomes of 1767 unique species (1.56% of all organisms in the database) encode methylsuccinate isomerases. Of these, 36,4% were part of gene clusters similar to the *C. necator* cluster, i.e., featuring the homologs of all five genes of its itaconate degradation cluster (Fig. 1c). In all other cases, at least one of these genes was absent, but genes encoding methylsuccinate isomerase were always located next to genes encoding an (*RS*)-methylsuccinyl-CoA dehydrogenase. Methylsuccinate isomerases were found almost exclusively in bacteria belonging to Pseudomonadota (96.2%), with 1235 methylsuccinate isomerases found in Betaproteobacteria (69.9%), 407 in Gammaproteobacteria (23.0%), and 78 in Alphaproteobacteria (0.04%) (Supplementary Table 3).

## Discussion

**Fig. 6:**
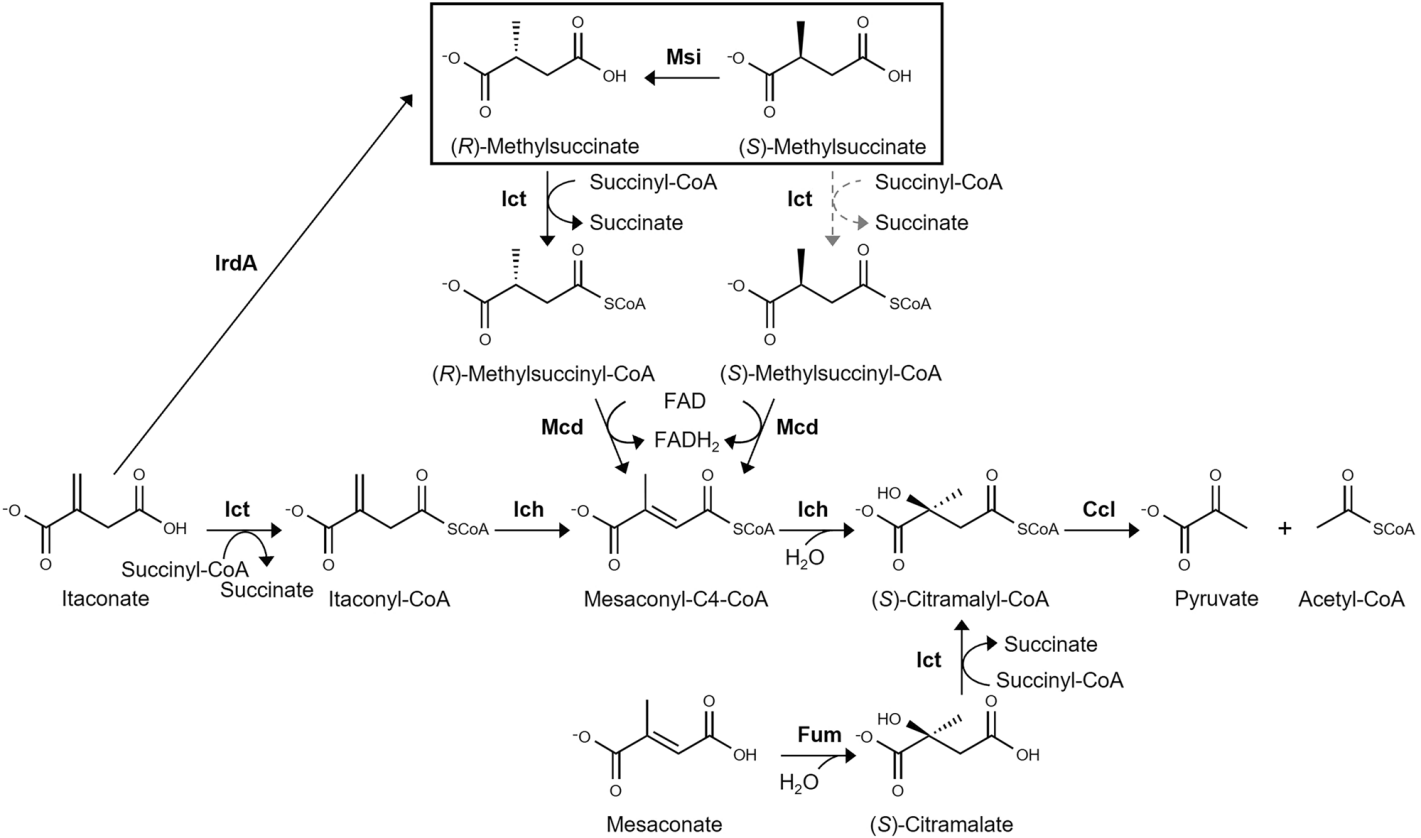
Metabolism of branched-chain C_5_-dicarboxylic acids in pseudomonads. Ict itaconate CoA transferase, Ich itaconyl-CoA hydratase, Ccl (*S*)-citramalyl-CoA lyase, Mcd (*RS*)-methylsuccinyl-C4-CoA dehydrogenase, Msi (*S*)-(*R*)-methylsuccinate isomerase, Fum class I fumarase catalyzing mesaconate hydratase reaction, IrdA itaconate-induced reductase reduces itaconate to methylsuccinate^31^. Gray dotted arrows represent a reaction that is unlikely to have occurred in vivo due to low efficiency of the itaconate CoA transferase with (*S*)-methylsuccinate.

There are currently two known sources of itaconate in ecosystems. Firstly, it is produced at high concentrations in the immune metabolism during macrophage activation^11–13^. Second, it is a fungal product produced by e.g., *Aspergillus* spp. or *Ustilago* spp^9,10,28,29^. Accordingly, the genes for itaconate degradation have been identified in many pathogens and saprophytic bacteria. In this study, we showed that the itaconate degradation gene cluster of many saprophytic bacteria (e.g., *Pseudomonas* spp., *Paraburkholderia* spp., *Cupriavidus* spp.) is also responsible for methylsuccinate degradation. In fact, this cluster can be considered more of a methylsuccinate degradation cluster, as all five of its genes are involved in methylsuccinate degradation, but only three of them are involved in itaconate metabolism (Fig. 6). In contrast, pathogenic species such as *Yersinia* spp. or *Brucella* spp. possess the itaconate degradation cluster consisting of only three genes that cannot metabolize methylsuccinate. The situation is even more complicated, as *Y. pestis*- and *P. aeruginosa*-like clusters are the products of convergent evolution (Fig. 1a, b). The *Y. pestis*-type cluster is predominant in pathogens and typically contains only three genes involved in itaconate degradation^20^. The *P. aeruginosa* cluster is found in both pathogens and saprophytic bacteria. In pathogens, it is usually a three-gene itaconate degradation cluster. In saprophytic bacteria, it is more likely to be a five-gene itaconate/methylsuccinate degradation cluster^20^. Note that the model organism *P. aeruginosa* is an opportunistic pathogen commonly found in soils.

There is biochemical indication that the *P. aeruginosa*-like cluster evolved to degrade both itaconate and methylsuccinate, whereas the *Y. pestis*-like cluster lacks methylsuccinyl-CoA dehydrogenase and methylsuccinate isomerase and is adapted for itaconate utilization/detoxification. Indeed, the catalytic efficiency of *Y. pestis* itaconate CoA transferase with methylsuccinate is ~30 times lower than with itaconate^20^, whereas the *P. aeruginosa* enzyme is similarly active with both substrates and the *C. necator* CoA transferase works better with methylsuccinate (Table 2). Furthermore, itaconyl-CoA hydratase has dual activity in the itaconate degradation pathway: as itaconyl-CoA isomerase and as mesaconyl-C4-CoA hydratase, although only the latter activity is relevant for methylsuccinate metabolism. In *Y. pestis*, the ratio of catalytic efficiencies of isomerase/hydratase and hydratase (0.53) is much higher than the corresponding ratio of the *P. aeruginosa* enzyme (0.09)^20^. This suggests a specific adaptation of the *Y. pestis* cluster to itaconate utilization alone, whereas the enzymes in saprophytic bacteria are also adapted to methylsuccinate degradation. This indicates the importance of methylsuccinate for soil ecosystems and of itaconate for pathogenicity.

While the source of itaconate in the environment is known, the origin of methylsuccinate remained unclear until recently. No natural pathway for the production of methylsuccinate was known, although a synthetic pathway via the reduction of citraconate has been established^30^. Just lately, Little et al.^31^ have shown that both itaconate and methylsuccinate are highly abundant in faecal samples from healthy humans, but not from antibiotic-treated patients. They found that methylsuccinate was produced by the reduction of itaconate by gut bacteria, and the corresponding itaconate-induced reductase (IrdA) has been identified in *Eggerthella lenta*^31^. Although there is no direct evidence for the “itaconate respiration” occurring in soils, this finding can be seen as proof of principle for the use of itaconate as an electron acceptor and for the formation of methylsuccinate in anaerobic environments. The stereoselectivity of itaconate-reducing enzyme in *E. lenta* has not been studied. Nevertheless, the existence of different reductases capable of reducing itaconate with different stereospecificity can be assumed, making the possibility of using both stereoisomers advantageous in the natural environment. Bacteria capable of fermenting itaconate are not yet known, and reduction of itaconate appears to be the main route for its conversion under anaerobic conditions. In contrast, itaconate produced during immunometabolism is unlikely to be reduced to methylsuccinate as the conditions are aerobic, explaining the redundancy of methylsuccinate utilization by pathogenic bacteria. A scheme summarizing the reactions known to be involved in the metabolism of C_5_-branched chain dicarboxylic acids is shown in Fig. 6.

The *P. aeruginosa* itaconate/methylsuccinate degradation cluster also encodes a gene for a putative ring-cleaving dioxygenase (Fig. 1). De Witt et al.^23^ proposed that it catalyses the conversion of 2-hydroxyparaconate to itatartarate and therefore acts as a 2-hydroxyparaconate lactonase. 2-Hydroxyparaconate is a fungal antimicrobial compound produced from itaconate that impairs the growth of various organisms^10,23^. This gene is only occasionally found in bacteria and is probably an adaptation to specific environmental conditions.

The only methylsuccinyl-CoA dehydrogenase identified so far was the enzyme involved in the ethylmalonyl-CoA pathway of acetyl-CoA assimilation^2,32,33^. However, this enzyme has a different stereospecificity and acts on a C1-CoA-ester of methylsuccinate (rather than a C4-ester) and is therefore a (2*S*)-methylsuccinyl-C1-CoA dehydrogenase. In the ethylmalonyl-CoA pathway, this enzyme produces mesaconyl-C1-CoA, which is further hydrated to β-methylmalyl-CoA and cleaved to propionyl-CoA and glyoxylate^2^. This module was used to design a synthetic CO_2_ fixation pathway, the CETCH cycle^34^, in which (2*S*)-methylsuccinyl-C1-CoA dehydrogenase was engineered towards oxidase activity by rational mutagenesis^34,35^. Our identification of (*RS*)-methylsuccinyl-C4-CoA dehydrogenase allows to connect the synthetic pathway with the module leading from methylsuccinate to mesaconyl-C4-CoA, (*S*)-citramalyl-CoA and finally to acetyl-CoA and pyruvate, contributing to the synthetic biology toolbox (Supplementary Fig. 3).

Similar to the (2*S*)-methylsuccinyl-C1-CoA dehydrogenase from the ethylmalonyl-CoA pathway, the (*RS*)-methylsuccinyl-C4-CoA dehydrogenase identified in this study belongs to the FAD-dependent acyl-CoA dehydrogenase family. Electrons from acyl-CoA dehydrogenases are usually transferred to the respiratory chain via electron transfer flavoproteins^36^, which we assume is also the case for the studied (*RS*)-methylsuccinyl-C4-CoA dehydrogenase, thus contributing to energy conservation via the respiratory chain.

**Fig. 7:**
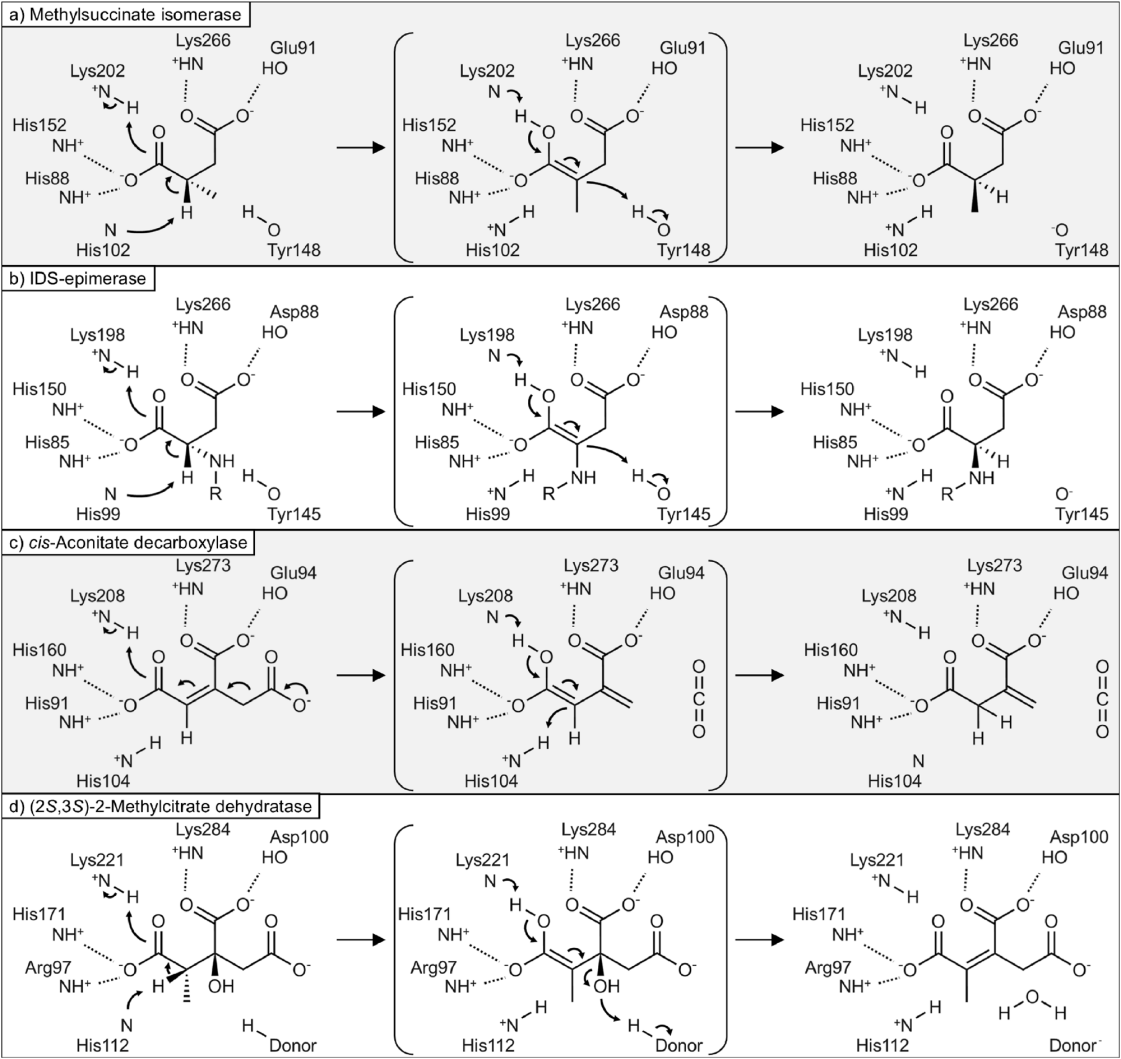
Proposed reaction mechanism for members of the MmgE/PrpD protein family. We propose that the reactions of methylsuccinate isomerase (**a**), IDS epimerase (**b**), *cis*-aconitate decarboxylase (**c**), and 2-methylcitrate dehydratase (**d**) all proceed through an enolate intermediate stabilized by one or both of the conserved lysine residues. For all reactions, the five conserved residues that coordinate the two carboxyl groups of the substrates and the catalytic histidine are shown. In addition, IDS epimerase and methylsuccinate isomerase have an additional catalytic tyrosine and catalyze their respective reactions via a two-base mechanism, whereas a one-base mechanism has been proposed for *cis*-aconitate decarboxylase^37,40^. In 2-methylcitrate dehydratase, the reaction proceeds either via a two-base mechanism, in which unknown proton donor donates the proton to the substrate, or via a one-base mechanism, in which the catalytic histidine donates the proton (abducted in the first step) back to the substrate.

The identification of the MmgE/PrpD family protein from the itaconate/methylsuccinate degradation cluster as an (*S*)-(*R*)-methylsuccinate isomerase extends our knowledge of the functional diversity of enzymes in this class. Enzymes of the MmgE/PrpD superfamily are found in a wide range of bacteria and archaea and in some eukaryotes, but not in plants. The proteins appear to be quite diverse and poorly characterized^37^. Characterized members of the MmgE/PrpD protein family are 2-methylcitrate dehydratase^38^, *cis*-aconitate decarboxylase in fungi and in animals^39,40^, iminodisuccinate (IDS) epimerase^37^ and now the (*S*)-(*R*)-methylsuccinate isomerase. Although the substrates of these enzymes (methylsuccinate, IDS, *cis*-aconitate, 2-methylcitrate) are quite different (Fig. 5b), they all share a four-carbon 1,4-dicarboxylate backbone with varying substitutions at the C2 and C3 positions. Furthermore, the reaction mechanisms, though different at first sight, share a common protonation/deprotonation of the C2 carbon (Fig. 7).

For the IDS epimerase reaction, a two-base mechanism with His99 and Tyr145 as the catalytic residues was proposed by Lohkamp et al.^37^. According to this mechanism (Fig. 7b), the abstraction of the α-proton facilitated by the adjacent carboxyl group results in an enolic intermediate that is stabilized by interactions with electrophilic groups of the enzyme. Similarly, in *cis*-aconitate decarboxylase, the conserved His103 was proposed to act as the catalytic residue, donating a proton to the C2 carbon in a one-base mechanism^40^. This conserved histidine is also present in 2-methylcitrate dehydratase (His104, 5MUX). Since the methylsuccinate isomerase reaction is similar to the isomerization in IDS-epimerase, and all putative catalytic residues from IDS-epimerase (H85, D88, H99, H150, K198, K266, PDB: 2HP0) are conserved in methylsuccinate isomerase (Fig. 5c), we propose the following mechanism (Fig. 7a). For the isomerization of (*S*)-methylsuccinate, His102 abducts a proton from the C2 methine group, resulting in the formation of a methylsuccinate enolate. The negatively charged enolate intermediate is stabilized by Lys266, which protonates the energy-rich dianion to form the methylsuccinate enol. Finally, Tyr148 donates the proton back to the C2 carbon, reintroducing the stereocenter and leading to the formation of (*R*)-methylsuccinate (Fig. 7a). In the reverse reaction (isomerization of (*R*)- to (*S*)-methylsuccinate), Tyr148 acts as the initial proton acceptor, while His102 acts as the proton donor. Since a similar mechanism could be suggested for the *cis*-aconitate decarboxylase reaction (Fig. 7c), and since the residues involved in the proposed stabilization of the enolate intermediate are conserved in the MmgE/PrpD family, we predict that all members of the MmgE/PrpD protein family have similar mechanism (Fig. 7). All the amino acid residues required for this mechanism are highly conserved in the MmgE/PrpD family (Fig. 5d). In silico docking simulation of *P. aeruginosa* methylsuccinate isomerase and (*R*)-methylsuccinate revealed that the carboxylic groups of the substrate are coordinated by these conserved amino acids (i.e., His88, Glu91, His102, His152, Lys202, and Lys266; Supplementary Fig. 4). Interestingly, the predicted structure suggests that the active site of methylsuccinate isomerase could also accommodate larger substrates with C3 modifications.

In summary, the results of our work demonstrate the importance of methylsuccinate degradation and extend our knowledge of the substrates used by bacteria in natural ecosystems.

## Materials and methods

### Materials

(*RS*)-Methylsuccinate was purchased from Sigma-Aldrich, (*R*)- and (*S*)-methylsuccinate from Apollo Scientific, (*RS*)-methylsuccinic anhydride from Sigma-Aldrich, (*R*)-citramalate from Sigma-Aldrich, and (*S*)-citramalate from Biosynth. Other chemicals and biochemicals were obtained from Sigma-Aldrich, Merck, Roth, VWR, or AppliChem. Materials for molecular biology were purchased from New England BioLabs. Materials and equipment for protein purification were obtained from GE Healthcare, Macherey-Nagel, or Millipore. Primers were synthesized by Sigma-Aldrich.

### CoA-ester synthesis

Acetyl-CoA, propionyl-CoA, butyryl-CoA, (*RS*)-methylsuccinyl-CoA, and succinyl-CoA were synthesized from the corresponding anhydrides and CoA according to Simon and Shemin^41^. Mesaconyl-C1-CoA and mesaconyl-C4-CoA were synthesized, as described in ref. ^20^.

(*R*)-Methylsuccinyl-CoA and (*S*)-methylsuccinyl-CoA were synthesized by incubating (10 °C) the CoA transferase from *C. necator* (0.5 mg protein ml^−1^) with (*R*)- or (*S*)-methylsuccinic acid (50 mM) and succinyl-CoA (5 mM) as the CoA donor in the reaction mixture (total volume 1 ml) containing 200 mM MOPS-KOH (pH 7.0), 5 mM DTT, and 5 mM MgCl_2_. The reaction was monitored by UHPLC and stopped after reaching equilibrium by adding 200 µl of 1 M formic acid, centrifuged (20,000 × *g*, 4 °C, 20 min), and the synthesized CoA-esters were purified by UHPLC.

### CoA-ester purification

The ACQUITY UPLC Premier-System H-Class (Waters, Milford, MA, USA) was used to purify the CoA-esters with a reversed-phase C18 column (InfinityLab Poroshell 120 EC-C18 2.1 × 50 mm, 1.9 μm, Agilent). The following acetonitrile gradient in 10 mM potassium phosphate buffer (pH 7.0) with a flow rate 0.55 ml min^−1^ was used: from 2 to 8% at 0–2.66 min; from 8 to 30% at 2.66–3.33 min; from 30 to 2% at 3.33–3.68 min; 2% at 3.68–5 min^42^. The column was heated to 25 °C. The corresponding peak was detected with the ACQUITY Premier eLambda PDA Detector and collected using the Waters Fraction Manager Analytical. The CoA-esters were lyophilized and stored at −20 °C until use.

The mass of the CoA-ester was determined with the Acquity QDa detector using the same program as described above, but with 10 mM formic acid buffer (pH 7.0) instead of potassium phosphate buffer. The Acquity QDa detector was used in positive electrospray ionization (ESI) mode and a TIC plot was measured between 400 and 900 m/z and SIR at 882 m/z with a cone voltage of 15 V. The probe temperature was set at 600 °C, the source temperature at 120 °C and the ESI capillary at 0.8 kV.

### Microbial strains and growth conditions

*C. necator* H16 (DSM 428) was obtained from the Deutsche Sammlung von Mikroorganismen und Zellkulturen. The cells were grown at 30 °C while shaking at 120 rpm in MSM medium^43^ containing 4.5 g l^−1^ Na_2_HPO_4_·2H_2_O, 1.5 g l^−1^ KH_2_PO_4_, 1.0 g l^−1^ NH_4_Cl, 0.2 g l^−1^ MgSO_4_·7H_2_O, 0.1 ml l^−1^ 100× SL6 trace element solution, and 1 ml l^−1^ iron-CaCl_2_ solution. 100× SL6 trace element solution contained 10.0 mg l^−1^ ZnSO_4_·7H_2_O, 3.0 mg l^−1^ MnCl_2_·4H_2_O, 30.0 mg l^−1^ H_3_BO_3_, 20.0 mg l^−1^ CoCl_2_·6H_2_O, 1.0 mg l^−1^ CuCl_2_·2H_2_O, 2.0 mg l^−1^ NiCl_2_·6H_2_O, 2.0 mg l^−1^ Na_2_MoO_4_·2H_2_O. Iron-CaCl_2_-solution contained 1.2 g l^−1^ ml Fe(III)-NH_4_-citrate (18% Fe-content) and 20 g l^−1^ CaCl_2_·2H_2_O. Succinate, itaconate, (*RS*)-methylsuccinate, (*R*)-methylsuccinate, or (*S*)-methylsuccinate were used as growth substrates at a final concentration of 20 mM.

*Escherichia coli* strains [Top10, BL21 (DE3)] were grown at 37 °C in lysogeny broth medium containing 10 g l^−1^ NaCl, 5 g l^−1^ yeast extract, and 10 g l^−1^ bacto-tryptone. Ampicillin was added to the cultures to a final concentration of 100 µg ml^−1^.

The cells were harvested by centrifugation (12,000 × *g*, 4 °C, 20 min) during the exponential growth phase, washed with saline solution [0.9% (w/v) NaCl], and stored at −20 °C until use.

### *C. necator* growth experiments

Growth experiments with *C. necator* were performed in the Epoch 2 microplate reader (BioTek, Winooski, VT, USA) using 24-well plates with a total volume of 1 ml per well. The optical density was followed by 600 nm. Cells grown on succinate were used as an inoculum for the growth experiments.

### *C. necator* cell extract preparation

For proteomic analysis, *C. necator* cells (100–150 mg) were suspended in 0.3 ml of 100 mM MOPS-KOH (pH 7.0) buffer in a 1.5 ml Eppendorf tube and disrupted using a Sonoplus HD 2070 ultrasonic disruptor (Bandelin, Berlin, Germany). The parameters used for sonification were: 80% amplitude, 1-s-pulse-1-s-pause-cycles, 1.5 min. The cell lysate was centrifuged twice (20,000 × *g*, 10 min, 4 °C), and the supernatant was frozen at −20 °C for proteomic analysis.

For cell extract assays, *C. necator* cells (2–5 g) were suspended in equal volume of 100 mM MOPS-KOH (pH 7.0) containing 0.1 mg ml^−1^ DNase I under anaerobic conditions. The cells were lysed using an HTU-Digi-French press (G. Heinemann, Schwäbisch Gmünd, Germany) at 14,000 psi under anaerobic conditions. The cell lysate was centrifuged twice (20,000 × *g*, 30 min, 4 °C) and stored anaerobically at −70 °C or used immediately for enzymatic assays.

### Gene cloning

For cloning, genes were amplified by Q5 High-Fidelity DNA Polymerase (NEB, Frankfurt, Germany) from genomic DNA of *C. necator*, *P. aeruginosa*, or *Pa. xenovorans*. The primers and annealing temperatures are summarized in Supplementary Table 4. The PCR conditions were as follows: 35 cycles of 10-s denaturation at 98 °C, 30-s primer annealing at temperature indicated in Supplementary Table 4, and elongation at 72 °C for 2 min. The PCR product was treated with the corresponding restriction enzymes (Supplementary Table 4), and ligated into pET16b for Bxe_B2582 and E6A55_22595 and in pET23b for E6A55_22600, using T4 DNA ligase (NEB). The plasmids were transformed into *E. coli* TOP10 for amplification.

The genes for heterologous expression of E6A55_22590, PA0881 and PA0879 were synthesized by BioCat (Heidelberg, Germany). The sequences were optimized for the expression in *E. coli* (Supplementary Table 2). For plasmid construction, the restriction sites NdeI and BamHI were added to the synthesized genes. The genes for E6A55_22590 and PA0881 were cloned into pET16b, whereas the gene encoding PA0879 was cloned into pET23a.

### Heterologous expression in *E. coli*

The amplified expression vectors were used to transform *E. coli* BL21 (DE3). The cells were grown at 37 °C in lysogeny broth medium with ampicillin (100 µg ml^−1^). Expression was induced at an optical density (OD_600_) of 0.5–0.8 with 0.5 mM isopropyl-ß-D-thiogalactopyranoside, and the temperature was lowered to 20 °C. The cells were harvested after overnight incubation and stored at −20 °C until use.

### Preparation of *E. coli* cell extracts

After heterologous expression, the cells were resuspended in equal volume of 20 mM Tris-HCl (pH 6.8) containing 0.1 mg ml^−1^ DNase I (for purification of E6A55_22600 and PA0879, 50 µM FAD was added) and lysed twice using a HTU-Digi-F-Press (G. Heinemann, Schwäbisch Gmünd, Germany) at 14,000 psi. The cell lysate was centrifuged twice (20,000 × *g*, 30 min, 4 °C), and the supernatant was used for enzyme purification.

### Enzyme purification

Enzyme purification was performed using gravity flow columns with Protino® Ni-NTA agarose (Macherey-Nagel, Düren, Germany). For the purification of E6A55_22595 and PA0882, 20 mM Tris-HCl (pH 6.8) was used for the equilibration of the column. For E6A55_22600 and PA0879, 50 µM of FAD was added to the equilibration and elution buffers. The unwanted proteins were washed of the column with 5-, 10-, 50-, and 150-mM imidazole before the desired His-Taq protein was eluted with 350 mM of imidazole. The same process was also applied for Bxe_B2582, E6A55_22590, and PA0881 purifications, but all buffers also contained 300 mM NaCl. The unwanted proteins were washed with 5-, 10-, 50-, 100-, and 200-mM imidazole, and the target protein was eluted with 400 mM imidazole. The enzymes were concentrated using Vivaspin® 20 (Sartorius, Göttingen, Germany) and the corresponding equilibration buffers. For the storage buffer for the dehydrogenases E6A55_22600 and PA0879, the FAD concentration was increased to 100 µM. The purified proteins were stored at 4 °C.

### Conversion of itaconate and methylsuccinate in cell extracts of *C. necator*

Succinyl-CoA-dependent conversions of itaconate, (*S*)-methylsuccinate, and (*R*)-methylsuccinate to acetyl-CoA by extracts of *C. necator* cells grown on itaconate, succinate, or (*RS*)-methylsuccinate were followed using UHPLC. The reaction mixture (total volume 100 µl) contained 100 mM MOPS-KOH (pH 7.0), 5 mM MgCl_2_, 5 mM DTT, and 5 mM substrate. The reaction was incubated in a thermomixer at 30 °C, 400 rpm. The reaction was started by the addition of 1 mM succinyl-CoA and at 0, 1, 3 min, 25 µl of the reaction mixture was transferred to ice and stopped with an equal volume of 1 M HCl containing 10% acetonitrile. The samples are centrifuged and analyzed by UHPLC.

### Enzyme assays

*CoA Transferase* activities of purified E6A55_22595 and PA0882 were measured using UHPLC^42^ at 30 °C in the reaction mixture (50 µl) containing 100 mM MOPS-KOH (pH 7.0), 5 mM MgCl_2_, 5 mM DTT, the corresponding CoA donor (succinyl-CoA, acetyl-CoA, propionyl-CoA, or butyryl-CoA, 1 mM) and CoA accepting carboxylate [itaconate, mesaconate, acetate, butyrate, propionate, (*S*)-methylsuccinate, (*R*)-methylsuccinate, (*S*)-citramalte, (*R*)-citramalate, glutarate, (*S*)-malate or (*R*)-malate, 10 mM] and enzyme. The reaction was typically stopped after 0 and 0.5 min by the addition of 1 M HCl/10% acetonitrile (sample:stop solution, 1:1 [v/v]), centrifuged, and analyzed by UHPLC (Poroshell column).

*Methylsuccinyl-CoA dehydrogenase* activity was measured using UHPLC (Poroshell column) at 30 °C in the reaction mixture containing 400 mM MOPS-KOH (pH 7.0), 5 mM MgCl_2_, 5 mM DTT, 1 mM PMS, 0.5 mM methylsuccinyl-CoA, and the enzyme. The formation of mesaconyl-CoA from (*R*)-, (*S*)-, or (*RS*)-methylsuccinyl-CoA was followed. The reaction was started by the addition of the enzyme and stopped after appropriate time intervals by the addition of 1 M HCl/10% acetonitrile (sample:stop solution, 1:1 [v/v]). The sample was centrifuged and analyzed by UHPLC. The activity of the enzyme was also tested with NAD(P) (1 mM) or with ferrecenium hexafluorophosphate (2.0 mM) instead of PMS, as described above. In addition, the activity of methylsuccinyl-CoA dehydrogenase was measured spectrophotometrically with cytochrome *c* in the reaction mixture containing 100 mM MOPS-KOH (pH 7.0), 5 mM MgCl_2_, 5 mM DTT, 1 mM (*RS*)-methylsuccinyl-CoA, 0.3 mM cytochrome *c,* and 0.5 mg protein ml^−1^ of the purified dehydrogenase from *C. necator* or *P. aeruginosa*. The reaction was followed at 550 nm for 5 min at 30 °C.

*Methylsuccinate epimerase* activity of MmgE/PrpD family proteins was measured using a coupled assay with the CoA transferase from *C. necator*. The epimerase was incubated for 1 min at 30 °C with 100 mM MOPS-KOH (pH 7.0), 5 mM MgCl_2_, 5 mM DTT, and 2.5 mM of the corresponding substrate [(*S*)-methylsuccinate, (*R*)-methylsuccinate, (*S*)-citramalate, (*R*)-citramalate]. Then, 1.5 mM succinyl-CoA and the *C. necator* CoA transferase (0.22 mg ml^−1^) were added to the reaction mixture, and it was incubated for 0.5 min. The reaction was stopped by the addition of 1 M HCl/10% acetonitrile (sample:stop solution, 1:1 [v/v]), centrifuged, and analyzed by UHPLC. Methylsuccinyl-CoA epimerase activity of MmgE/PrpD family proteins was tested similarly to methylsuccinate epimerase activity, but methylsuccinate and succinyl-CoA were replaced by (*S*)-methylsuccinyl-CoA (0.5 mM) and succinate (100 mM) and the formation of succinyl-CoA was followed. The epimerase activity of MmgE/PrpD family proteins with itaconate, citraconate, mesaconate, *cis*-aconitate, and *trans*-aconitate was measured using UHPLC with a Bluebird column. The reaction mixture contained 100 mM MOPS-KOH (pH 7.0), 5 mM MgCl_2_, 5 mM DTT, and 10 mM of the corresponding substrate [itaconate, citraconate, mesaconate, *cis*-aconitate, *trans*-aconitate] and 0.7 mg protein ml^−1^ of the purified MmgE/PrpD family protein from *C. necator*. The mixture was incubated for 5 min at 30 °C before the reaction was stopped by the addition of 1 M HCl/10% acetonitrile (sample:stop solution, 1:1 [v/v]), centrifuged, and analyzed by UHPLC.

To determine (*R*)-(*S*)-malate isomerase activity, a spectrophotometric assay was performed using citrate synthase and malate dehydrogenase as coupling enzymes to monitor the formation of NADH at 365 nm. The reaction mixture (total volume 400 µl) contained 100 mM MOPS-KOH (pH 7.0), 5 mM MgCl_2_, 5 mM DTT, 1 mM NAD, 10 mM (*R*)-malate or (*S*)-malate, 0.6 mM acetyl-CoA, 0.5 U citrate synthase, 5 U malate dehydrogenase, and 0.7 mg/ml purified MmgE/PrpD family protein from *C. necator*. The reaction was monitored at 30 °C for 5 min.

### Analytical UHPLC

CoA and CoA-esters were detected with Agilent 1290 Infinity II UHPLC using a reversed-phase C18 column (Agilent InfinityLab Poroshell 120 EC-C18 1.9 μm 2.1 × 50 mm column), as described in ref. ^42^.

The following acetonitrile gradient in 10 mM potassium phosphate buffer (pH 7.0) with a flow rate 0.55 ml min^−1^ was used: from 2 to 8% at 0–2.66 min; from 8 to 30% at 2.66–3.33 min; from 30 to 2% at 3.33–3.68 min; 2% at 3.68–5 min^42^. Retention times were: succinyl-CoA, 1.0 min; CoA, 1.15 min; mesaconyl-C1-CoA, 1.20 min; mesaconyl-C4-CoA, 1.40 min; methylsuccinyl-CoA, 1.44 min; acetyl-CoA, 2.0 min; propionyl-CoA, 2.6 min; butyryl-CoA, 3.5 min. In this method, the CoA-esters formed double peaks with the second, smaller peak being 0.15 min away from the first peak. Reaction products and standard compounds were detected by UV absorbance at 260 nm with a 1290 Infinity II diode array detector (Agilent), and the amount of product was calculated from the relative peak area. The identification of the CoA-esters was based on co-chromatography with standards and analysis of the UV spectra of the products. The specific activities were calculated by considering the peaks of consumed and formed CoA-esters. The concentration of the latter was calculated by multiplying the starting substrate concentration by the relative abundance [%] of the formed CoA-ester (integrated peak area).

Organic acids were detected with Agilent 1290 Infinity II UHPLC using a Nucleoshell Bluebird reversed-phase C18 column (Macherey-Nagel 150/2 EC 2.7 µm column). The analysis was performed at 40 °C with a 50 mM phosphate buffer (pH 2.5) and an isocratic flow (0.400 ml min^−1^) for 7 min. Reaction products and standard compounds were detected by UV absorbance at 210 nm with a 1290 Infinity II diode array detector (Agilent).

### Proteomics

The relative quantification of the cell lysate proteomes of *C. necator* was achieved with data-independent label-free high-definition mass spectrometry (HDMS) protein expression analysis on Synapt G2 Si following filter-based tryptic digestion using the UniProt *C. necator* database^44^. In brief, the proteins in cell lysates were reduced, alkylated and tryptically digested on 10 kDa centrifugal filter devices and prepared for reversed-phase liquid chromatography coupled to HDMS at 250 ng/μl in 0.1% formic acid and 5% acetonitrile. For statistical analyses, Progenesis QIP software was used (nonlinear diagnostics/Waters Corp., Manchester, UK; fixed modification carbamidomethylation, variable modification methionine oxidation, 1 missed cleavage allowed).

### Hidden-Markov-model (HMM) creation and validation

Using manually validated seed sequences for methylsuccinate isomerase from *P. aeruginosa*, *P. fluorescence*, *C. necator*, and *Pa. xenovorans*, homologues were extracted from NCBI-nr using the online implementation of BLASTP (https://blast.ncbi.nlm.nih.gov/, accessed January 20, 2025)^45^, using an *E*-value threshold of 1e-5. The initial hits were further reduced by manually checking the gene neighborhoods to determine when the first false positives were recovered and consequently removing all hits with weaker *E*-values than these false positives. The corresponding amino acid sequences were aligned using MAFFT^46^. Alignments were loaded into Seaview^47^ for manual inspection and edge trimming to remove low occupancy positions at the edges. The HMM was built using hmmbuild^48^. To evaluate its performance, we used hmmsearch^48^ with an *E*-value threshold of 1e-5 to identify homologous sequences in the Genome Taxonomy Database (GTDB) r220^26^. We plotted the score distribution as a function of the *E*-value-based rank, and then evaluated at which score the off-target homologous sequences were recovered.

While the methylsuccinate isomerases from *P. aeruginosa*, *P. fluorescence*, *C. necator*, and *Pa. xenovorans* all achieved sequence scores above 750, there was a significant number of hits that were not part of complete clusters, as seen in the above organisms, but were adjacent to methylsuccinate dehydrogenases. Since we have shown that these two enzymes can link methylsuccinate degradation to itaconate degradation, the colocalization of genes encoding these two enzymes strongly suggests that the MmgE/PrpD homolog is indeed a methylsuccinate isomerase. We therefore chose a cutoff of 550, which includes not only hits in complete degradation clusters, but also these “incomplete” gene clusters. To test the applicability of the HMMs (and their score thresholds) to other databases, we tested them on UniProt, UniRef100, and UniRef90^47,49,50^, which show similar patterns of distribution of score vs. *E*-value rank to the GTDB (Supplementary Fig. 5, Supplementary Data 3).

To assess the distribution of methylsuccinate isomerases across prokaryotic diversity, we used the GTDB r220^26^. This database contains all prokaryotic genomes recovered so far, dereplicated to the species level, and thus allows to assess the distribution of these gene families across the prokaryotic tree of life. The concatenated proteins of all genomes represented in release 220 of this database were searched for methylsuccinate isomerases using hmmsearch^48^ with the HMM and threshold defined above.

### Phylogenetic analysis

Multiple sequence alignment was performed using MAFFT with default settings^46^. Phylogenetic trees were inferred using the maximum likelihood method implemented in IQ-TREE (v2.4)^51^. Model selection was performed automatically using IQ-TREE’s ModelFinder, and branch support was evaluated with 1000 bootstrap replicates to assess the robustness of the inferred topology. The resulting phylogenetic tree was visualized and annotated using the Interactive Tree of Life (iTOL) online tool (https://itol.embl.de/).

### Protein structure prediction

The three-dimensional structure of the target protein was predicted using AlphaFold2^52^ via the AlphaFold Protein Structure Prediction Server (https://alphafoldserver.com/). The full-length amino acid sequence was submitted using default settings consistent with those reported in the AlphaFold 3 publication^53^. Template structures were automatically selected from the Protein Data Bank (PDB) using a cutoff date of September 30, 2021. Multiple sequence alignments were generated using a combination of genetic databases as described in ref. ^53^. Model confidence was assessed based on predicted Local Distance Difference Test scores provided by the server. The predicted structure was visualized and analyzed using UCSF ChimeraX (v1.9)^54^.

### Docking simulation

Molecular docking was performed using Webina (v1.0.5), a web-based implementation of AutoDock Vina^55^ that enables ligand-receptor docking entirely within a web browser environment^56,57^. The target protein structure was prepared by removing water molecules and heteroatoms, followed by the addition of hydrogen atoms where necessary. Docking was performed with all conformers of methylsuccinate. All input files were formatted in PDBQT using the built-in preparation utilities available within Webina. A grid box was defined to encompass the known or predicted active/binding site of the target receptor. The docking parameters were set to default values unless specified otherwise, with an exhaustiveness value of 8 to ensure adequate sampling of ligand conformations. Docking simulations produced multiple binding poses ranked by predicted binding affinity (kcal/mol) and the best-scoring pose for each ligand was selected for further analysis. Docking results were visualized and analyzed using UCSF ChimeraX (v1.9)^54^.

### Other methods

DNA sequence determination of purified plasmids was performed by Microsynth (Göttingen, Germany), using Sanger sequencing. Protein concentration was measured according to the Bradford method^58^ using BSA as a standard. Sodium dodecyl sulfate-polyacrylamide gel electrophoresis (SDS-PAGE; 12.5%) was performed according to ref. ^59^. Proteins were visualized using Coomassie blue staining^60^. *K*_M_ and *V*_max_ values were calculated by GraphPad Prism 5 software.

### Statistics and reproducibility

The data values are represented as mean ± SD of the replicates from two (growth experiments), three (proteomics), or three or more (enzyme assays) independent experiments. The obtained replicates were in good agreement with each other. SD, *V*_max_, and *K*_M_ values were calculated using GraphPad PRISM software. Statistical analysis of proteomic data was performed using Progenesis QIP software.

### Reporting summary

Further information on research design is available in the Nature Portfolio Reporting Summary linked to this article.

## Supplementary information

Reporting Summary

Description of Additional Supplementary Files

Supplementary Information

Supplementary Data 1

Supplementary Data 2

Supplementary Data 3

Supplementary Data 4

## Data Availability

All data supporting the findings are available within the article and/or its supplementary materials. The raw data are presented in the paper and/or available at the Miami server of the University of Münster, doi: 10.17879/42968649463^61^ and at the ProteomeXchange server (PXD065931). For any further inquiries about the work please contact the corresponding author.
