# Supplementary material for: Pseudomonadal itaconate degradation gene cluster encodes enzymes for methylsuccinate utilization
Source: Commun Biol. 2025 Jul 24;8:1099. doi: 10.1038/s42003-025-08538-2 (PMC12290011; doi:10.1038/s42003-025-08538-2)
Supplement: Supplementary file 2 — Description of Additional Supplementary Files [file 42003_2025_8538_MOESM2_ESM.docx]

Description of Additional Supplementary Files

**File name:** Supplementary Data 1

**Description:** Comparative proteomics, itaconategrown cells versus succinate-grown cells.

**File name:** Supplementary Data 2

**Description:** Comparative proteomics, methylsuccinate-grown cells versus succinategrown cells.

**File name:** Supplementary Data 3

**Description:** Source data for Fig. S2.

**File name:** Supplementary Data 4

**Description:** Source data for Fig. S5.
