# Supplementary material for: Pseudomonadal itaconate degradation gene cluster encodes enzymes for methylsuccinate utilization
Source: Commun Biol. 2025 Jul 24;8:1099. doi: 10.1038/s42003-025-08538-2 (PMC12290011; doi:10.1038/s42003-025-08538-2)
Supplement: Supplementary file 3 — Supplementary Information [file 42003_2025_8538_MOESM3_ESM.pdf]

Pseudomonadal itaconate degradation gene cluster encodes enzymes for methylsuccinate utilization

Lena Gonner[1], Eric A. Cassens[1], Simone König[2], Ivan A. Berg[1]*

[1] Institute for Molecular Microbiology and Biotechnology, University of Münster, Münster, Germany
[2] Core Unit Proteomics, Interdisciplinary Center for Clinical Research, Medical Faculty, University of Münster, Münster, Germany
*Corresponding author (ivan.berg@uni-muenster.de)

Supplementary data

Supplementary Table 1. Expression of enzymes involved in metabolism of itaconate and other C_5-branched chain dicarboxylic acids in *C. necator* grown on different substrates. For the full data sets, please see **Supplementary Data 1** and **2**. MeSuc, (*RS*)-methylsuccinate.

**Enzyme**	**Accession No (E6A55_)**	**Unique peptides**	**Fold differences during growth on the following substrate (in compare to succinate)**	
			**Itaconate**	**MeSuc**
Methylsuccinyl-CoA dehydrogenase	Q0K3E4 (_22600)	11	83.19	3.55
Itaconate CoA transferase	Q0K3E5 (_22595)	1	Infinity	Infinity
(*S*)-(*R*)-Methylsuccinate isomerase	Q0K3E6 (_22590)	16	2.26	1.18
(*S*)-Citramalyl-CoA lyase	Q0K3E7 (_22585)	14	1.41	1.63
Itaconyl-CoA hydratase	Q0K3E8 (_22580)	12	3.67	1.83

Supplemetary Table 2. Synthesized genes. The optimised nucleotide sequences for the genes synthesised by BioCat are listed below, together with the corresponding vector.

C. necator H16 (*S*)-(*R*)-methylsuccinate isomerase E6A55_22590 (in pET16b)

ATGAGCCAACCCGCAGACACCACCTACCCCACCCGCCAGCTTTGCGACTTCCTGGCCGACCTCAAGCTGGCCGACGTG
CCCGCGCCCGTGATCGAGCGCACCAAGGACCTGTTCCTGGACTGGATCGCCTCGGCCATCGCCGGCAAGGATGCCCC
CGCCGTGCGCAAGCTGCAGGAGTTCGCCGCGGCCATGGGCCCGGACCAAGGCGGCGCCGAAGTGCTGGTGGACCGC
CGCCGCACCTCGCCCTACTTTGCCGCGCTGATCAACGGCGCCTCCTCGCACGTGGTCGAGCAGGACGACGTGCACAA
CGGCTCGGTGCTGCACCCGGCCGCGGTGGTGTTCCCTGCCGTGGTGGCAGCCGCGCAGACCGAGGGCAAGACCGGC
GCCGAAGTGCTGCTGGCCTCGATCGCCGGCTATGAGGCCGGCATCCGCATCGGCGAATTCATGGGCCGTTCGCACTA
CCGCGTGTTCCACACCACCGGCACGGTGGGCACGCTGGCCGCCGCCGCGGCCGTGGCCAAGCTCTACGGCCTCGAT
GCCGAAGGCATCAACCAGGCACTGGGCTCGGCCGGCACGCAGGCCGCGGGCCTGTGGGAATTCCTGCGCGACGCCG
CCGACTCCAAGCAGCTGCATACCGCCAAGGCCGCGGCCGATGGCCTGCAGTCAGCCTGGCTCGCGCGCGCCGGCTTC
ACCGGCGCGAAGCAGATCCTGGAAGGCGCGCAGGGCATGGCCGCCGGCATGTCCAGCGATGCCAACCCCGCCTGCC
TGACCGACGGCCTGGGCACGCGCTGGGCCACCGCCGAGACCTCGTTCAAGTTCTTCGCCTCATGCCGCCACACGCAC
CCGGCCGCCGATGCGCTCAAGGCGCTGATGCAGCGCGAAGGCATCGGCGCCGACCAGGTCGCCAGCGTCACCACGC
ACGTGCACCAGGGCGCCATCGACGTGCTCGGCCCGGTGGTCAACCCCACCACCATCCACCAGGCCAAGTTCTCGATG
GGCACGGTGCTGGGGCTGGTGGCCGTGCACGGCCATGCGGGTCTGGGCGAGTTCGAGCAGCATGCGCTGCAGGACC
CGAAGGTGACCGCCTTCCGCGGCAAGGTCGGCATGGAACTGGATGCCGAGATCAACGCCGCCTACCCGCGCCAGTGG
ATCGGCCGCGTTACCGTCACGACCACCGACGGCCGCACACTGGCGGCGCGCGTCGACGTGCCCAAGGGCGATCCCG
ACAACACGCTGTCGCGCCCGGAACTGGAAGCGAAGGCGCTGCAGCTGGGCGCGTTCCGCCACGGCGCGAGCGAGGC
CGAGATGCGCGCCATCATCCAGCGCGTGTGGGCGCTGGAGCAGGCCCCGAACGTCAACGACTGGCTGCCCGCCGCA
GGCTGA

P. aeruginosa (*S*)-(*R*)-methylsuccinate isomerase PAO1 PA0881 (in pET16b)

ATGACCGACTACACCCAGCAACTCGCCGGCTTTCTCGCCGGCCTGCGCTACCAGGACCTGCCGCCGGCGGTGCTGGC
GCGGATGGAAGAATTGTTCCTCGACTGGCTCGGCTCGGCGCTGGCCGGCAAGGGCCAGCATCCGATCCCATTGTTCGA
GCGCTACGCCGAACGCATGGGCCCGGCCGACGGTAGCGCGCAGATCCTGCCCAGCCGGCGGCGCAGCTCGCCGTAT
TTCGCCGCCCTGGTGAACGGCGCGGCTTCCCACGTGGTGGAGCAGGACGACCTGCACAACAGCTCGGTGCTGCACCC
CGCGGCGGTGGTCTTCCCTGCTGCCCTGGCCGCGGCCCAGGACCTCGGCCGCAGCGGCGCCGAGCTGATCCTCGCG
GCGGTGGCCGGCTACGAGGCCGGCATCCGCATCGGCGAATTCCTCGGCCGTTCCCACTACCGGGTGTTCCATACCACT
GCCACGGTCGGCACCCTCGCCGCGGCAGTGGCGGTGGGCAAGCTGATGGACTTCGACCGCGAGCGCTTCGTCGACCT
GCTCGGCAGCGCCGGAACCCAGGCCGCCGGGCTCTGGGAGTTCCTCCGCGACGCCGCCGATTCCAAGCAGTTGCATA
CCGCCAAGGCCGCCGCCGACGGGCTGCTCGCCGCCTATCTCACCGCCGACGGCCTGAGCGGCGCGCGACACATCCT
CGAAGGCGAGCAGGGGATGGCCGCCGGGATGTCCAGCGACGCCGACCCCGAGCGCCTGGTGGATCGCCTCGGCAGC
CGCTGGGCGTTGCTGGAAACCTCGTTCAAGTTCCACGCCTCGTGCCGCCATACCCATCCCGCGGCCGACGCGCTGCTG
GCGCTGATGCAGCGCGAAGGGCTCGATCACTCGCAGATCGCCGCGGTCACCGCGCGGGTCCACCAGGGCGCCATCG
ACGTGCTCGGCCGGGTGGTCGAGCCGCAGACCGTGCACCAGGCGAAGTTCTCCATGGGCACCGTGCTGGGCCTGATC
GCCGTGTACGGCAAGGCCGGCCTCGGCGAGTTCCATCGGCATGCGCTGAGCGACCCGCGGGTCGCAGCGTTCCGCG
AGCGGGTCGAGATGCGCCTGGACCCGGAGGTCGACGCCGCCTATCCGCAGCGCTGGCTCGGGCGGGTCGAAGTACT
GGATCGCGAAGGGCGGCGCCATACCGCCGCCATCGACGAACCCAAGGGCGATCCCGGCAATACCCTCAGCCGCGACG
AACTGGCCGACAAGTTCCGCCGCCTGCTGGCGTTCTCCGGCGCGGCCACCGACGCCGAGGCGGAAATCCTTATCCAG
CGCGCCTGGGGCCTGCGCCAGGCGCCTTCCGTAGCGCCGCTGATCTGA

P. aeruginosa methylsuccinyl-CoA dehydrogenase PAO1 PA0879 (in pET23a)

ATGAATCCGAACAGCGAAGAGCTGAACGCGATCCGCGAGGGCGTACGTGCCCTCTGCGCCGAATTCGACGCCGCCTA
CTGGCGCCGCATCGATGAGCAGCGCGGTTTTCCCGAGGCCTTCGTCAAGGCCCTGACCGACGCCGGCTGGCTCTCGG
CGATGATCCCGGAGGAGTACGGCGGTTCCGGTCTGGGCCTGGCCGAGGCCTCGGTGATCCTGGAGGAAGTCAACCGC
TGCGGCGGCAACTCCGGCACCGTGCACGGGCAGATGTACAACATGTTCACGTTGCTGCGTAACGGCAGCGAGGCGCA
GAAGCGCTTCTACCTGCCGAAGCTGGCCAGCGGCGAGCTGCGCCTGCAGTCGATGGGGGTGACCGAGCCGACCACCG
GCACCGATACCACCAAGATCAAGACCACCGCCGTGCGCAAGGGCGACCGCTATGTGATCAACGGGCAGAAGGTGTGG
ATCTCGCGGATCCAGCACTCCGACCTGATGATCCTGCTGGCGCGCACCACGCCGCTGGCCGAGGTGAAGCGCAAGTC
CGAAGGCATGTCGATCTTCCTCGTCGACCTGCGCGAGGCCATCGGCAAGGGCCTGACCGTGCAGCCGATCGCCAACAT
GGTCAACCACGAGACCAACGAGCTGTTCTTCGACAACCTGGAGATCCCCGCCGATAGCCTGATCGGCGAGGAAGGCAA
GGGCTTCCGCTACATCCTCGACGGCCTCAATGCCGAGCGCACCCTGATCGCCGCCGAATGCATCGGCGACGGCCGCT
GGTTCATCGACAAGGCCAGCCACTATGCCCGCGACCGCGTGGTCTTCGGCCGGCCGATAGGGCAGAACCAGGGCGTG
CAGTTCCCCATCGCCGAGGCGCACATCGAAGTGGAGGCCGCCGACCTGATGCGCTGGCGCGCCTGCCAGGAGTACGA
CGCCGGGCTCAACGCCGGGGCCAGCGCGAACATGGCCAAGTACCTGGCGGCGAAAGCTTCCTGGGAGGCGGCCAAC
GCCTGCCTGCAGACCCACGGCGGCTTCGGCTTCGCCTGCGAGTACGACGTCGAGCGCAAGTTCCGCGAGACCCGCCT
GTACCAGGTGGCGCCGATCTCCACCAACCTGATCCTGTCCTACGTGGCCGAGCACCTGCTCGAACTGCCGCGCTCGTT
C

Supplementary Table 3. Phylogeny of prokaryotes containing methylsuccinate isomerase encoding genes in their genomes, as identified using GTDB with hmmsearch. The number of identified representatives is given next to the name of the taxon. Organisms shown feature methylsuccinate isomerase with a sequence score >550. Please note that the taxonomic names shown here are according to the GTDB taxonomy, in which Betaproteobacteria are considered to be part of Gammaproteobacteria.

**Bacteria**					
**Phylum**		**Class**		**Order**	
Pseudomonadota	1720	Gammaproteobacteria	1642	Burkholderiales	1235
Actinomycetota	14	Alphaproteobacteria	78	Pseudomonadales	365
Bacillota	6	Rubrobacteria	14	Rhizobiales	63
Chloroflexota	6	Gemmatimonadetes	6	Arenicellales	21
Gemmatimonadota	6	Myxococcia	6	Rubrobacterales	14
Myxococcota	6	Sulfobacillia	6	Rhodobacterales	8
Acidobacteriota	2	Ktedonobacteria	4	Gemmatimonadales	6
Desulfobacterota	2	Chloroflexia	2	Myxococcales	6
Desulfobacterota	1	Desulfuromonadia	2	Nevskiales	6
Desulfobacterota	1	Terriglobia	2	Sulfobacillales	6
Planctomycetota	1	Binatia	1	Cardiobacteriales	4
		Deferrimicrobia	1	Ktedonobacterales	4
		Planctomycetia	1	Nitrococcales	4
				Caulobacterales	3
				Granulosicoccales	3
				Desulfuromonadales	2
				Dongiales	2
				Sphingomonadales	2
				Bryobacterales	1
				Chloroflexales	1
				Deferrimicrobiales	1
				JAHELT01	1
				Pirellulales	1
				Steroidobacterales	1
				Thermomicrobiales	1
				UBA7540	1
				UBA9968	1
				XJ16	1
				Xanthomonadales	1
**Archaea**					
**Phylum**		**Class**		**Order**	
Thermoplasmatota	2	Thermoplasmata	2	Thermoplasmatales	2

Supplementary Table 4. PCR Primers used for gene amplification.

Oligonucleotide	Sequence	Restriction enzyme	Annealing temp. in °C
Bxe_B2582_fwd	5´-TGAGTGCATATGAACGATCATCCCAGTCT-3´	NdeI	50
Bxe_B2582_rev	5´-GATGACAAGCTTACGAACAGGTAGGAGCG-3´	HindIII	
E6A55_22595_fw	5´-AAAACATATGTGCATGTCCCATTC-3´	NdeI	58
E6A55_22595_rev	5´-AAAAGGATCCGTCGTGTGTTC-3´	BamHI	
E6A55_22600_fw	5´-AAAACATATGATTCAATCCGAGCG-3´	NdeI	62
E6A55_22600_rev	5´-AAAAGCTTGAACGAACGCGG-3´	HindIII	

Supplementary Table 5. Accession numbers for the sequences used for the construction of the phylogenetic trees shown on the **Figure 5**.

**Fungal *cis*-aconitate decarboxylase**	
**Organism**	**Accesion (NCBI)**
Aspergillus terreus	Q0C8L3
Aspergillus alliaceus	XP_031894140.1
Aspergillus leporis	KAB8072425.1
Claussenomyces sp. TS43310	KAI9735751.1
Aspergillus tanneri	XP_033428713.1
Talaromyces pinophilus	KAF3406875.1
Loxospora ochrophaea	MCJ1376373.1
Aspergillus pseudocaelatus	KAE8410274.1
Aspergillus caelatus	XP_031925694.1
Aspergillus pseudotamarii	XP_031917697.1

**Mamalian *cis*-aconitate decarboxylase**	
**Organism**	**Accesion (NCBI)**
Canis lupus	XP_038287133.1
Bos taurus	XP_005213930.2
Sus scrofa	XP_005658501.2
Ursus maritimus	XP_008684878.2
Panthera tigris	XP_015399241.1
Mus musculus	6R6T
Homo sapiens	6R6U
Gorilla gorilla gorilla	XP_004054663.5
Vombatus ursinus	XP_027700400.1
Ornithorhynchus anatinus	XP_028913960.1

**2-Methylcitrate dehydratase**	
**Organism**	**Accesion (NCBI)**
Salmonella enterica	5MVI
Escherichia coli	1SZQ
Bacillus subtilis	5MUX
Pseudomonas aeruginosa	6S62
Yarrowia lipolytica	Q6C354
Mycobacterium tuberculosis	O06582
Cupriavidus necator	Q937N6
Corynebacterium glutamicum	Q8NSL3
Salmonella typhimurium	P74840
Saccharomyces cerevisiae	Q12428

***Bacillus* IRG1 homologs**	
**Organism**	**Accesion (NCBI)**
Bacillus subtilis	7BRA
Bacillus inaquosorum	WP_268281678.1
Bacillus mojavensis	WP_412854244.1
Bacillus atrophaeus	WP_268369163.1
Bacillus amyloliquefaciens	WP_353638901.1
Bacillus cabrialesii	WP_215797935.1
Bacillus velezensis	WP_251257322.1
Bacillus siamensis	WP_328221329.1
Bacillus safensis	WP_262090502.1
Bacillus [Multispecies]	WP_105928068.1

**IDS-Epimerase**	
**Organism**	**Accesion (NCBI)**
Agrobacterium tumefaciens	2HP3

**Methylsuccinate isomerase**	
***P. fluorescence* cluster**	
**Organism (according to GTDB)**	**Accesion (GTDB)**
Pseudomonas_E fluorescens_BQ	NZ_CABVGX010000060.1_13
Pseudomonas_E fulva_B	NC_015556.1_2631
Pseudomonas_E sp001945445	NZ_MSXW01000054.1_16
Pseudomonas_E daroniae	NZ_QJUI01000010.1_12
Pseudomonas_E dryadis	NZ_QJUM01000005.1_210
Pseudomonas_E fulva_D	NZ_CP082928.1_64
Pseudomonas_E seleniipraecipitans	NZ_FNBM01000009.1_22
Pseudomonas_E straminea	NZ_FOMO01000002.1_26
Pseudomonas_E punonensis	NZ_FRBQ01000002.1_622
Pseudomonas_E fulva_A	NZ_JACBYV010000001.1_1009

***P. aeruginosa* cluster**	
**Organism (according to GTDB)**	**Accesion (GTDB)**
Pseudomonas aeruginosa	NZ_LN831024.1_4184
Pseudomonas_E psychrophila	NZ_JYKZ01000005.1_54
Stutzerimonas urumqiensis	NZ_RBZQ01000001.1_232
Pseudomonas_E saxonica	NZ_VFIO01000013.1_27
Pseudomonas_E helleri_A	NZ_WIWC01000007.1_50
Stutzerimonas stutzeri_R	NZ_CP046902.1_3405
Pseudomonas_E sp019345465	NZ_JAHKRC010000022.1_41
Pseudomonas_E bubulae	NZ_UYJA01000001.1_708
Pseudomonas_E fragi_D	NZ_NQKQ01000013.1_76
Pseudomonas_H gallinarum	NZ_JACSQG010000009.1_93

***Pa. xenovorans* cluster**	
**Organism (according to GTDB)**	**Accesion (GTDB)**
Paraburkholderia xenovorans	NC_007952.1_444
Burkholderia cepacia_C	NZ_LDWR01000002.1_25
Burkholderia pyrrocinia_D	NZ_LOZU01000013.1_85
Paraburkholderia sp004339745	NZ_SMGP01000005.1_375
Burkholderia sp004343775	NZ_PTXL01000003.1_542
Paraburkholderia aromaticivorans_A	NZ_CP051515.1_2918
Paraburkholderia sp028283785	NZ_JAPWKB010000014.1_278
Burkholderia cenocepacia	NZ_UAQZ01000008.1_21
Paraburkholderia sp900996235	NZ_CAAJGM010000263.1_177
Burkholderia sp902832865	NZ_CADFDL010000002.1_413

***C. necator* cluster**	
**Organism (according to GTDB)**	**Accesion (GTDB)**
Cupriavidus necator	NC_015723.1_622
Cupriavidus basilensis_D	NZ_AHJE01000044.1_198
Cupriavidus neocaledonicus	NZ_AQUR01000107.1_435
Cupriavidus necator_B	NZ_SROX01000015.1_108
Cupriavidus sp020618415	NZ_CP085345.1_868
Cupriavidus sp023952475	NZ_JALHRX010000004.1_338
Cupriavidus alkaliphilus	NZ_FMAD01000005.1_75
Cupriavidus taiwanensis_B	NZ_LT984804.1_587
Cupriavidus yeoncheonensis	NZ_CAJPUY010000008.1_96
Cupriavidus numazuensis	NZ_CAJPVI010000034.1_48

***A. kashmirensis* Cluster**	
**Organism (according to GTDB)**	**Accesion (GTDB)**
Advenella kashmirensis	NC_017964.1_4941
Oligella ureolytica	NZ_KB892296.1_26
Advenella kashmirensis_A	NZ_KI650979.1_1116
Advenella mimigardefordensis	NZ_CP003915.1_4294
Advenella sp002810445	NZ_NEXS01000001.1_235

Advenella_A faeciporci	NZ_BMYS01000034.1_13
Advenella sp003533245	NZ_JABUXU010000004.1_37
Advenella_A mandrilli	NZ_JAENGP010000002.1_289
Advenella_A alkanexedens	NZ_JAHSPR010000003.1_320
Advenella incenata	NZ_SHKO01000002.1_108

M. qingshengii cluster

Organism (according to GTDB)	Accesion (GTDB)
Mesorhizobium qingshengii	NZ_FMXM01000015.1_70
Mesorhizobium sp000427725	NZ_ATYO01000009.1_199
Mesorhizobium sp000502875	NZ_AYWX01000002.1_224
Mesorhizobium ciceri_B	NZ_JAFG01000005.1_171
Mesorhizobium loti	NZ_QGGH01000001.1_1134
Mesorhizobium kowhaii	NZ_MZXV01000051.1_332
Mesorhizobium huakuii_A	NZ_CP050296.1_6413
Mesorhizobium ciceri	NZ_JARIYC010000010.1_142
Mesorhizobium opportunistum	NC_015675.1_2785
Mesorhizobium loti_G	CP050293.1_814

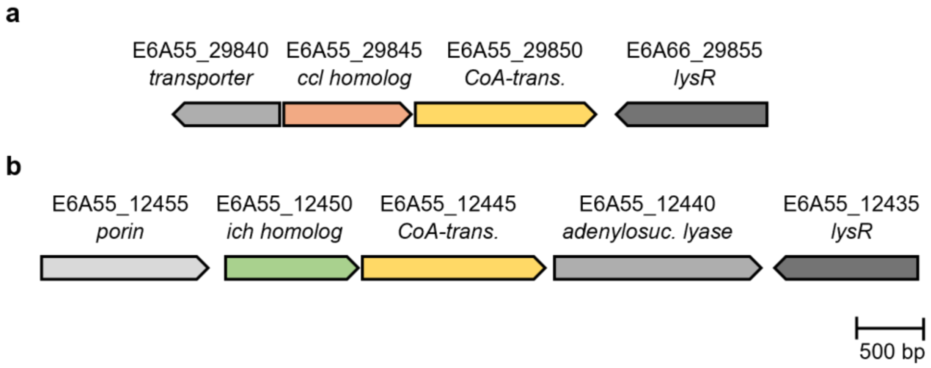

Supplementary Figure 1. Gene cluster containing genes for (A) second (*S*)-citramalyl-CoA lyase (ccl) homolog (E6A55_29845, 48/56% identity/similarity to PA0883), putative glycine/betaine ABC transporter (E6A55_29840), putative CoA transferase (E6A55_29850), and putative LysR transcription regulator (29855), **and for (B) second itaconyl-CoA hydratase homolog** (E6A55_12450, 48/58% identity/similarity to PA0878), porin (E6A55_12455), putative CoA transferase (E6A55_12445), putative adenylosuccinate lyase family protein (E6A55_12440), and putative LysR transcription regulator (E6A55_12435).

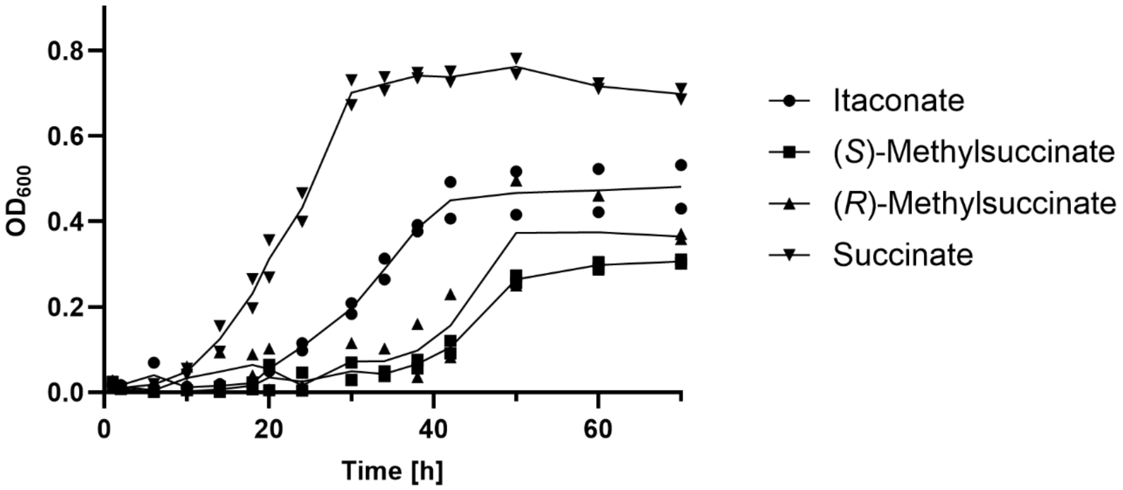

Supplementary Figure 2. Growth of *C. necator* on different substrates. Growth from a culture previously grown on succinate. The experiment was performed in duplicates, with the error bars representing the standard deviation. Numerical source data are in **Supplementary Data 3**.

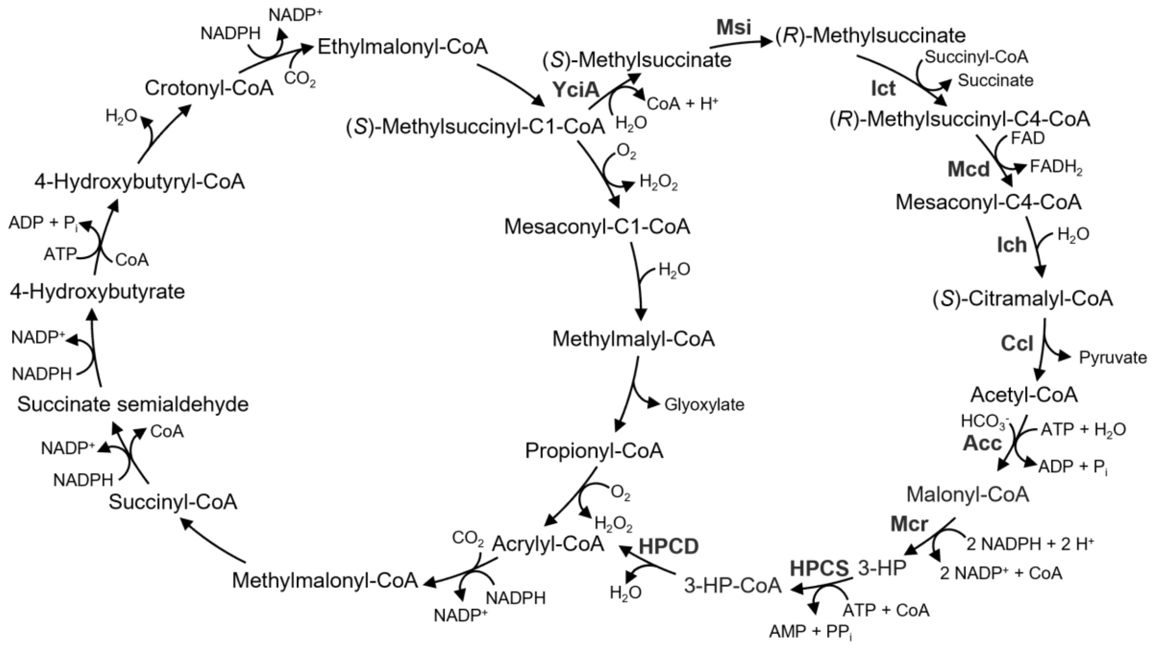

Supplementary Figure 3. Potential pathway that involves the identified (*RS*)-methylsuccinyl-C4-CoA dehydrogenase linking the synthetic CETCH cycle[1] to acetyl-CoA and pyruvate.

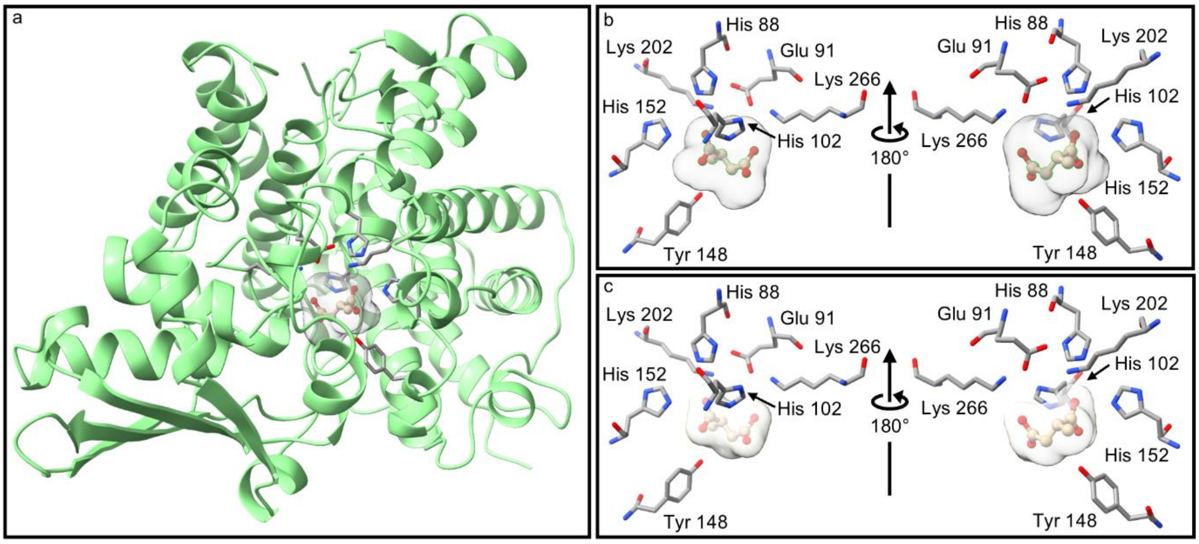

Supplementary Figure 4. Predicted structure (a) and active site (b, c) of *P. aeruginosa* methylsuccinate isomerase docked to (*R*)-methylsuccinate. The predicted structure showed the typical fold described previously for other members of the MmgE/PrpD family[2,3]. The active site and substrate surfaces are shown in (b) and (c), respectively. Amino acids conserved in all MmgE/PrpD family members that coordinate the two carboxyl groups of the substrate, as well as the catalytic His102 and Tyr148 are shown. The active site of the protein was considerably larger than the substrate, suggesting that this enzyme may also utilize larger substrates.

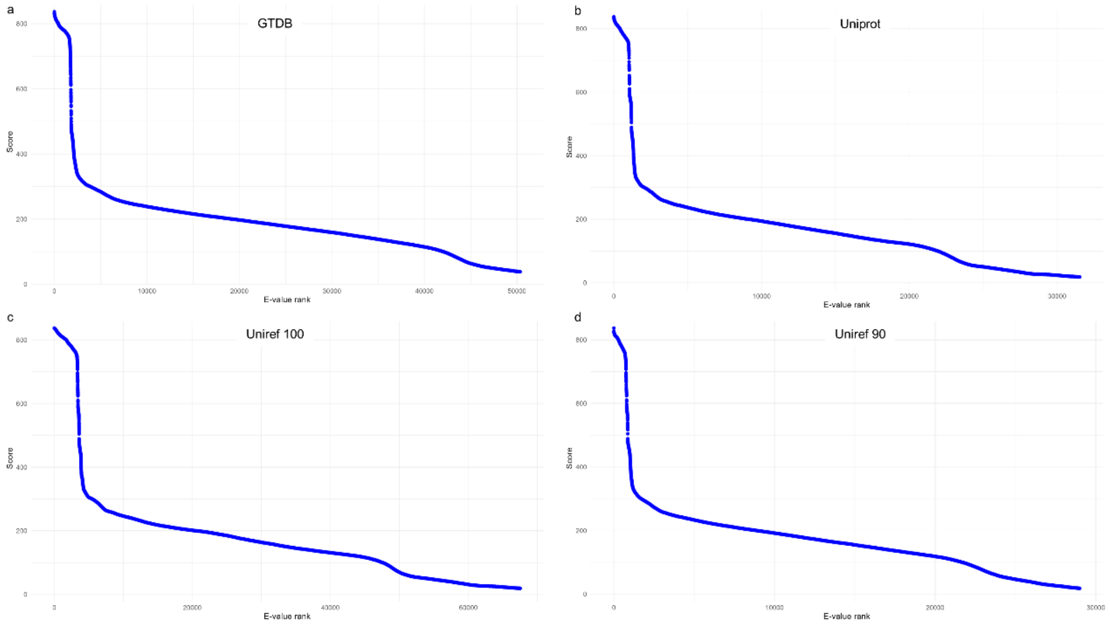

Supplementary Figure 5. Score vs E-value rank plot of HMM hits from different databases. To evaluate the applicability of the HMM, we tested it on UniProt (**b**), UniRef100 (**c**) and UniRef90 (**d**), showing similar patterns of distribution of score vs E-value rank to the GTDB (**a**). Numerical source data are in **Supplementary Data File 4**.

Supplementary References

1. Schwander, T. et al. A synthetic pathway for the fixation of carbon dioxide in vitro. *Science* **354**, 900-904 (2016). doi: 10.1126/science.aah5237.
2. Lohkamp, B., Bäuerle, B., Rieger, P. G. & Schneider, G. Three-dimensional structure of iminodisuccinate epimerase defines the fold of the MmgE/PrpD protein family. *J. Mol. Biol.* **362**, 555-566 (2006). DOI: 10.1016/j.jmb.2006.07.051.
3. Chen, F. et al. Crystal structure of *cis*-aconitate decarboxylase reveals the impact of naturally occurring human mutations on itaconate synthesis. *Proc. Natl. Acad. Sci. USA.* **116**, 20644-20654 (2019). doi: 10.1073/pnas.1908770116.
